# Stage-dependent effects of cognitive reserve on memory and brain structural integrity across the spectrum from healthy aging to Alzheimer’s disease

**DOI:** 10.3389/fnhum.2026.1830181

**Published:** 2026-07-15

**Authors:** Laura Serra, Sabrina Bonarota, Giulia Caruso, Matteo Mancini, Sofia Sperati, Carlotta Di Domenico, Federico Maria Tamigi, Francesco Di Lorenzo, Francesco Ricci, Carlo Caltagirone, Giacomo Koch, Federico Giove, Marco Bozzali, Laura Petrosini

**Affiliations:** 1 Department of Human Sciences, Università degli Studi Guglielmo Marconi, Rome, Italy; 2 Neuroimaging Laboratory, Fondazione Santa Lucia IRCCS, Rome, Italy; 3 Museo Storico della Fisica e Centro Studi e Ricerche Enrico Fermi, Rome, Italy; 4 Department of Psychology, Sapienza University of Rome, Rome, Italy; 5 Experimental Neuropsychophysiology Laboratory, Fondazione Santa Lucia IRCCS, Rome, Italy; 6 Scientific Direction, Fondazione Santa Lucia IRCCS, Rome, Italy; 7 Department of Neuroscience and Rehabilitation, University of Ferrara, Ferrara, Italy; 8 Department of Neuroscience “Rita Levi Montalcini”, University of Turin, Turin, Italy; 9 SC Neurologia 2U, AOU Città della Salute e della Scienza, Turin, Italy; 10 Laboratory of Experimental and Behavioral Neurophysiology, Fondazione Santa Lucia, Rome, Italy

**Keywords:** aging, Alzheimer’s disease, cognitive performance, cognitive reserve, mild cognitive impairment, source-based morphometry, structural brain networks

## Abstract

**Introduction:**

Cognitive reserve (CR) has been proposed as a key factor explaining inter-individual variability in cognitive performance despite comparable neuropathology. However, its role across the Alzheimer’s disease (AD) continuum remains unclear. This study investigates stage-dependent effects of CR on the relationship between memory performance and brain structural network integrity across healthy subjects (HS), individuals with subjective cognitive decline (SCD), and patients with amnestic mild cognitive impairment (a-MCI), and AD dementia.

**Materials and methods:**

A total of 209 participants underwent a comprehensive neuropsychological assessment and 3T MRI. Source-based morphometry identified three grey matter structural covariance networks, involving orbitofrontal-temporal-insular regions (OTIN), precuneus-posterior cingulate cortex (PreCiN), and cingulate-hippocampal regions (CHiN). A composite memory score was derived using factor analysis. Regression and moderation models examined the predictive and moderating effects of CR (operationalized as years of education) and network integrity on cognitive performance within each group.

**Results:**

OTIN and PreCiN showed progressive structural vulnerability along the AD continuum, whereas CHiN showed no significant between-group differences. Across the sample, OTIN and PreCiN integrity significantly predicted cognitive performance. In HS, CR was positively associated with memory performance independently of structural network integrity, suggesting an additive protective role of cognitive reserve in healthy aging. In the SCD group, CR was not directly associated with memory, and only limited effects emerged, indicating early alterations in reserve-related processes. In a-MCI patients, the significant interaction between CR and OTIN integrity suggested patterns consistent with compensatory mechanisms, with higher reserve supporting memory despite structural decline. In AD patients, CR and its interaction with structural networks no longer predicted cognitive outcomes, suggesting a possible exhaustion of reserve capacity.

**Conclusion:**

These findings support a stage-dependent model of CR, characterized by an additive protective role in healthy aging, patterns consistent with compensatory recruitment in early cognitive decline, and a possible loss of reserve effectiveness beyond a critical neuropathological threshold. Distinct network vulnerabilities and stage-specific CR effects highlight potential windows for reserve-enhancing interventions across the AD continuum.

## 1 Introduction

The concept of cognitive reserve (CR) has gained prominence as a framework for understanding why individuals with similar levels of neuropathology may exhibit markedly different cognitive abilities (Stern, 2012; Boyle et al., 2013; Stern et al., 2020). CR reflects the accumulation of cognitive and neural resources throughout the lifespan (Stern, 2002; Stern, 2009; Stern, 2012) and is commonly estimated using proxies such as educational attainment, occupational complexity, or participation in cognitively stimulating activities (Stern, 2009; Arenaza-Urquijo et al., 2017; Pettigrew and Soldan, 2019; Serra et al., 2018; Pappalettera et al., 2024; Pinto et al., 2024). In healthy aging, CR appears to support the ongoing recruitment of efficient neural networks, helping to maintain cognitive performance (Barulli and Stern, 2013; Cabeza et al., 2018; Serra et al., 2026). In contrast, during preclinical stages of dementia, including mild cognitive impairment (MCI) and, possibly, subjective cognitive decline (SCD), CR may reflect the engagement of the brain’s compensatory mechanisms in response to emerging pathology and declining neural efficiency. In later stages of Alzheimer’s disease (AD), the potential protective effects of CR appear to diminish (Stern, 2012; Stern et al., 2020; Serra and Gelfo, 2019; Serra et al., 2026). In this framework, we hypothesize that CR is not an invariant resource but rather a changing capacity whose effectiveness may depend, at different clinical stages, on the integrity and resilience of neural substrates, as well as interactions with demographic, genetic, brain-related factors and disease stage (Stern, 2009; Tucker and Stern, 2011; Nelson et al., 2022; Cordeiro et al., 2024). Understanding these stage-specific variations is essential for developing models of cognitive aging that account for both inter-individual variability and the mechanisms underlying resilience. Key brain regions supporting memory and executive functions, including the temporal lobes, precuneus, cingulate gyrus, and dorsal and orbitofrontal prefrontal cortices, are likely central to CR mechanisms (Solé-Padullés et al., 2009; Serra et al., 2017; Lee et al., 2019; Pettigrew and Soldan, 2019). The integrity of these regions plays a central role in normal cognitive functioning, and their disruption occurs in AD brains since early clinical stages (Migliaccio and Cacciamani, 2022; Jones and Graff-Radford, 2021; Dadario and Sughrue, 2023). It remains to be clarified what role these brain structures play in individuals with subjective cognitive complaints. The present study addressed this gap by focusing on the stage-dependent role of CR across the continuum from healthy aging to clinical AD. CR was operationalized as years of formal education. Education represents the most widely validated and consistently used proxy for CR in aging and dementia research, given its robust and reproducible association with brain resilience and delayed cognitive decline across the lifespan (Stern, 2012; Serra et al., 2018; Dufouil et al., 2003). It has been demonstrated that individuals with higher levels of education are more likely to engage in cognitively stimulating activities across the lifespan, which are associated with neuroplasticity mechanisms underlying CR and a delayed clinical manifestation of neurodegenerative processes (Katzman, 1993; Vance and Crowe, 2006). Moreover, education is a well-standardized measure that can be reliably and comparably assessed across participants, making it particularly suited for the retrospective characterization of CR in clinical samples.

Based on this theoretical framework and prior evidence, we formulated the following stage-specific hypotheses: in HS, consistent with the neural efficiency hypothesis (Stern, 2009; Barulli and Stern, 2013) and with previous evidence from our group showing that education does not act as a significant moderator in cognitively healthy individuals (Serra et al., 2026), we expected CR to directly and independently support memory performance, reflecting the efficient use of cognitive resources accumulated over the lifespan rather than active compensation for structural loss. In SCD individuals, based on emerging evidence suggesting subtle alterations in reserve-related neural processes despite preserved objective performance (Jessen et al., 2014), and consistent with our previous findings that reserve mechanisms begin to change even at this earliest preclinical stage (Serra et al., 2024; Serra et al., 2026), we hypothesized that the protective relationship between CR and cognition would begin to weaken, reflecting an early disruption of reserve mechanisms before detectable structural changes. In a-MCI patients, where neuropathological burden is increasing but has not yet overwhelmed neural resources, and consistent with both the compensation-related utilization of neural circuits hypothesis (Reuter-Lorenz and Cappell, 2008; Stern, 2009) and our previous evidence of significant interactions between CR and lifelong stimulating activities at this stage (Serra et al., 2024; Serra et al., 2026), we anticipated that CR would actively moderate the relationship between structural network integrity and memory performance. Finally, in AD patients, and consistent with the concept of a neuropathological tipping point beyond which compensatory mechanisms are overwhelmed (Jack et al., 2013; Stern et al., 2020), and with the absence of significant CR moderation effects observed in our previous behavioral study (Serra et al., 2026), we expected CR to lose its modulatory role entirely.

Specifically, we investigated here the modulating role of CR in the relationship between structural network integrity and cognitive performance, and we explored whether this effect varies across different clinical stages of AD. By focusing on stage-specific moderation, this study attempts to provide new insights into how CR’s potential protective effects may change as pathology progresses, offering an understanding of its more gradual role in both healthy and pathological cognitive aging.

## 2 Materials and methods

### 2.1 Participants

The study sample included 209 individuals consecutively recruited at the Memory Clinic of Fondazione Santa Lucia IRCCS (Rome, Italy). The sample is divided in 57 patients with AD (mean ± SD years of age: 73.2 ± 6.9; mean ± SD years of education 10.0 ± 4.4; sex F/M = 38/19; mean ± SD MMSE score: 21.4 ± 4.0), 59 with amnestic MCI (a-MCI) (mean ± SD years of age: 72.2 ± 8.2; mean ± SD years of education 12.8 ± 4.5; sex F/M = 32/27; mean ± SD MMSE score: 26.8 ± 2.0), 44 individuals with subjective cognitive decline (SCD) (mean ± SD years of age: 68.2 ± 7.1; mean ± SD years of education 13.0 ± 3.4; sex F/M = 27/17; mean ± SD MMSE score: 27.5 ± 3.7), and 49 cognitively healthy elderly (Healthy Subjects, HS) (mean ± SD years of age: 64.6 ± 8.1; mean ± SD years of education 14.3 ± 3.9; sex F/M = 31/18; mean ± SD MMSE score: 27.5 ± 4.4).

Participants with AD and a-MCI, but not those in the SCD or HS groups, received a neuro-biologically confirmed diagnosis of AD, supported by cerebrospinal fluid biomarkers according to the Core 1 Research Framework (Jack et al., 2024). Clinically, all a-MCI patients were classified as single-domain amnestic MCI based on established criteria (Albert et al., 2011). By definition, these individuals did not meet the criteria for major neurocognitive disorder; their Clinical Dementia Rating scores (Hughes et al., 1982) did not exceed 0.5, and their Mini-Mental State Examination scores (Folstein et al., 1975; Magni et al., 1996) were within the normal range (≥23.8).

SCD participants met the inclusion criteria proposed by Jessen et al. (2014, 2020), including: (i) self-reported memory complaints affecting daily functioning; (ii) absence of objective impairment in memory or other cognitive domains on standardized neuropsychological evaluation; and (iii) absence of alternative medical or psychiatric explanations for the reported symptoms. HS were required to report no subjective cognitive concerns, perform within normal ranges across all cognitive domains on formal testing, in the absence of any significant medial temporal lobe atrophy.

Exclusion criteria for all participants included a Hachinski Ischemic Score greater than 4 (Hachinski et al., 1975). Major systemic or neurological diseases, psychiatric disorders, and other neurological conditions were carefully investigated by neurologists and ruled out in all participants. Finally, in SCD individuals and HS subclinical psychological symptoms (e.g., depression and anxiety) were clinically assessed by trained psychologists and ruled out.

Only right-handed individuals, as determined by the Edinburgh Handedness Inventory (Büsch et al., 2010), were included.

The study protocol was approved by the Ethics Committee of Fondazione Santa Lucia IRCCS (approval number CE/PROG.856 23-07-20). All participants, or their legal representatives, provided written informed consent before the enrolment. Procedures adhered to the principles of the Declaration of Helsinki and its subsequent amendments, in line with international ethical standards.

### 2.2 Neuropsychological assessment

All participants completed a comprehensive neuropsychological battery assessing multiple cognitive domains. Verbal episodic long-term memory was evaluated using the 15-Word List Test with immediate, 15 min delayed recall and recognition task from which can be derived two different and independent scores: hit rates (W-HR: the number of words correctly identified on 15 items previously studied) and false alarms (W-F: the number of distractor words incorrectly identified on 30 new items), as well as the Short Story Test (immediate and 20-min delayed recall) (Carlesimo et al., 1996, 2002). Visuospatial episodic long-term memory was assessed with the Rey-Osterrieth Complex Figure Test, including immediate and 20 min delayed recall (Carlesimo et al., 1996). Short-term memory and working memory were measured using the Digit Span and the Corsi Block-Tapping Task, both administered in forward and backward conditions (Monaco et al., 2013). Executive functions were evaluated using the Phonological Word Fluency Test (Carlesimo et al., 1996) and the Modified Card Sorting Test (Nocentini et al., 2002). Language abilities were assessed with the Object Naming subtest of the BADA (Batteria per l’Analisi dei Deficit Afasici; Battery for the Analysis of Aphasic Deficits) (Miceli et al., 1991). Reasoning abilities were measured using Raven’s Colored Progressive Matrices (Carlesimo et al., 1996). Constructional praxis was evaluated through the copy of simple drawings, the copy of drawings with landmarks (Carlesimo et al., 1996), and the copy condition of the Rey-Osterrieth Complex Figure (Carlesimo et al., 2002).

All neuropsychological measures were administered according to standardized procedures. For the purposes of the present study, raw neuropsychological scores were not adjusted for age, sex, or education. Instead, these demographic variables were included as covariates of no interest in the statistical analyses.

### 2.3 MRI procedure

MRI data were collected on a 3T MAGNETOM Prisma scanner (Siemens Healthcare, Erlangen, Germany), equipped with a 64-channel head–neck coil. For each participant, the following whole-brain sequences were collected: (a) T2-weighted turbo spin-echo (TSE) (matrix = 448 × 448; field of view [FOV] = 220 × 220 mm^2^, 29 axial slices, slice thickness = 4 mm; repetition time [TR] = 3,490 ms, echo time [TE] = 95 ms; acquisition time = 2 min 42 s; (b) fluid-attenuated inversion recovery (FLAIR) (matrix = 240 × 256; FOV = 240 × 256 mm^2^; 176 sagittal slices; slice thickness = 1 mm; TR = 8,000 ms; TE = 314 ms; inversion time [TI] = 2,350 ms; acquisition time = 7 min 54 s); and (c) a T1-weighted multi-echo MPRAGE (MEMPRAGE) sequence (matrix = 256 × 256; FOV = 256 × 256 mm^2^; 176 sagittal slices; slice thickness = 1 mm; TR = 2,500 ms; TE = 1.67/3.48/5.29/7.10 ms; TI = 1,080 ms; flip angle = 8 °; acquisition time = 7 min 27 s).

### 2.4 MRI data processing

Group differences in grey matter (GM) organization were investigated using source-based morphometry (SBM), a multivariate, data-driven approach for identifying patterns of structural covariance (Xu et al., 2009). Unlike voxel-based morphometry (VBM) (Ashburner and Friston, 2000), which evaluates each voxel independently, SBM is a multivariate approach that applies independent component analysis (ICA) (McKeown and Sejnowski, 1998) to T1-weighted images to extract spatial components representing coordinated variations in grey matter (GM) (Gupta et al., 2019). Through this decomposition, SBM identifies groups of brain regions that show similar patterns of structural variability across individuals, thereby capturing the network-level organization of GM. While VBM provides voxel-wise estimates of regional GM volume, SBM focuses on covariance among spatially distributed regions, offering a complementary perspective on large-scale structural architecture (Xu et al., 2009; Gupta et al., 2019).

In this framework, while medial temporal lobe atrophy was clinically assessed through visual inspection (see point 2.6) to ensure the diagnostic representativeness of the clinical cohort, the primary quantitative objective of our study was to move beyond isolated regional measurements. By employing SBM, we aimed to characterize coordinated patterns of structural reorganization across the whole brain, providing a more comprehensive understanding of grey matter covariance networks rather than focusing solely on *a priori* expected regional volume loss.

#### 2.4.1 Data preprocessing

Preprocessing of T1-weighted images was performed in SPM12 (Wellcome Center for Human Neuroimaging, London, United Kingdom). Prior to any processing step, a rigorous visual quality control was independently performed on all raw T1-weighted anatomical images slice-by-slice to detect potential motion-related artifacts (e.g., ringing, blurring, or ghosting), severe susceptibility artifacts, or gross structural abnormalities. Preprocessing included four steps. First, images were segmented into GM, white matter (WM), and cerebrospinal fluid (CSF). Second, GM maps were spatially normalized to the Montreal Neurological Institute (MNI) template using the unified segmentation-normalization algorithm. Third, normalized images were modulated to preserve information about local GM volume. Finally, images were smoothed using an isotropic Gaussian kernel with a full width at half maximum (FWHM) of 8 mm. All normalized GM maps were visually re-checked to ensure precise anatomical alignment, ruled out geometric distortions, and verified the absence of systematic segmentation errors. The resulting pre-processed GM maps were then entered into the subsequent SBM-ICA analysis.

#### 2.4.2 SBM ICA analysis

SBM analysis was performed using the Group ICA of fMRI Toolbox (GIFT) v4.0c^1^. The dataset was decomposed into 20 independent components, with the number of components estimated using the minimum description length (MDL) criterion (Li et al., 2007). Independent components were extracted using the Infomax algorithm, which maximizes statistical independence between components by minimizing shared information, and identifying distinct spatial patterns of GM variation across participants (Bell and Sejnowski, 1995; Lee et al., 1999). The stability of the decomposition was evaluated using the ICASSO toolbox, with 20 repetitions in RandInit mode. Component reliability was quantified using the quality index (Iq), which ranges from 0 to 1 and reflects the reproducibility of each component (Himberg et al., 2004).

The ICA decomposition generated participant-specific loading coefficients, reflecting the degree to which each individual expressed the spatial GM pattern associated with a given component. These values were derived from the mixing matrix produced by the ICA algorithm, organized as a participant × component matrix, where each row corresponds to a single participant and each column represents an independent component. Higher loading values indicate stronger expression of the corresponding structural network in a given individual. The spatial pattern associated with each component was represented by voxel-wise weights reflecting grey matter concentration (GMC). To resolve the inherent sign-indeterminacy of spatial ICA, the spatial component maps were automatically scaled and flipped based on data skewness within the GIFT toolbox to ensure positive weights for voxels heavily contributing to each network. This sign-convention framework renders the subject-specific loading coefficients sign-invariant; because the data are mean-centered during preprocessing, these loadings index the relative expression of structural covariance. Consequently, lower or negative loading values indicate a relative reduction in GMC within the corresponding network compared to the sample average, whereas higher loading values reflect a relatively higher expression or preservation of the structural network. Participant-specific loading coefficients were used as variables of interest in subsequent group comparisons, correlation analyses, regression models, and moderation analyses.

In parallel, the ICA algorithm generated a source matrix describing the voxel-wise spatial distribution of each independent component. Each row of this matrix corresponds to a spatial pattern defined by a set of voxel weights mapping the distribution of the component across the brain. These spatial maps represent coherent patterns of structural covariance that may vary in their expression across individuals. Component maps were thresholded, converted into 3D volumes, and rendered as GM structural networks for visualization.

Following ICA decomposition, the 20 extracted components were visually inspected (see Supplementary Figures S1–S20), and those that included brain regions defined *a priori* as most relevant to the aims of the present study (the temporal lobes, precuneus, cingulate gyrus, and dorsal and orbitofrontal prefrontal cortices, the key brain regions supporting memory and executive functions, as mentioned in the Introduction) were retained for further analyses. In particular, the selection of these specific components followed a rigorous two-step validation protocol:

- First (Hypothesis-Driven Identification of data-driven structural components): we specifically targeted networks encompassing regions of interest defined a priori as above detailed;
- Second (Anatomical Refinement and Quality Control): each of the 20 components was meticulously inspected using the orthogonal viewer in GIFT to confirm its neuroanatomical validity. Some components represented anatomically coherent structural networks, characterized by clusters of voxels localized within functionally related brain regions. In contrast, some other components exhibited fragmented or peripheral patterns, primarily representing stochastic noise or motion-related artifacts. The emergence of either neuro-biologically relevant signals or artefactual components is an expected outcome and a standard feature of ICA-based procedures. In this framework, we selected only the neuro-biologically relevant components (see Supplementary Figures S1–S20).

The distinction between artefactual and neurobiologically meaningful components was based on a systematic two-step validation protocol applied to all 20 extracted components, as illustrated in Supplementary Figures S1–S20. In the first step (anatomical coherence evaluation), each component was assessed for the spatial contiguity and neuroanatomical plausibility of its voxel-wise weight distribution overlapping the spatial maps on the Ch2bet template available in the Group ICA of fMRI Toolbox (GIFT). Biologically meaningful components were characterized by spatially coherent clusters of voxels localized within functionally related grey matter regions, consistent with known large-scale structural brain networks. Their spatial patterns showed smooth, symmetric, and anatomically recognizable distributions across cortical and subcortical grey matter territories. In the second step (artifact identification), components were flagged as artefactual when their spatial patterns displayed one or more of the following features: (i) fragmented or spatially discontinuous distributions lacking neuroanatomical coherence; (ii) predominant localization in non-grey matter regions, such as ventricular borders, white matter tracts, or skull-adjacent areas; (iii) patterns consistent with known sources of MRI artifacts, including head motion, magnetic field inhomogeneities, or susceptibility effects; or (iv) bilateral symmetric rim-like patterns at the brain edges, typically indicative of normalization or segmentation residuals rather than true structural variance. Only components satisfying the criteria for neuroanatomical coherence and free from artefactual features were retained for subsequent analyses. This dual-criterion approach is consistent with established procedures for quality control in ICA-based structural neuroimaging studies (Xu et al., 2009; Gupta et al., 2019; Beckmann and Smith, 2004) and ensures that the retained components reflect genuine patterns of grey matter covariance rather than noise-driven decomposition artifacts.

### 2.5 Medial temporal lobe atrophy

Hippocampal atrophy was assessed on T1-weighted images using the Medial Temporal Lobe Atrophy (MTA) scale (Scheltens et al., 1992; Pereira et al., 2014). This visual rating scale ranges from 0 (no atrophy) to 4 (severe atrophy), with scores ≥1.5 considered indicative of clinically significant medial temporal lobe degeneration. MTA scores were obtained bilaterally and then averaged across hemispheres to derive a single composite index of medial temporal lobe atrophy for each participant.

### 2.6 Statistical analyses

SPSS-25.0^2^ was used for statistical comparisons. AD, a-MCI, SCD, and HS groups were compared on demographic variables and cognitive performance (neuropsychological battery) using one-way ANOVAs (Supplementary material for statistical results on the neuropsychological battery tests).

To derive a unified memory index, Principal Component Analysis (PCA) was conducted on six memory measures: 15-Word List Immediate Recall (W-IR), 15-Word List Delayed Recall (W-DR), 15-Word List Recognition Hit Rates (W-HR), 15-Word List Recognition False Alarms (W-F), Short Story Immediate Recall (SS-IR), and Short Story Delayed Recall (SS-DR). Crucially, Hit Rates and False Alarms were entered as independent variables to allow the PCA to determine their respective weights. This approach accounts for recognition performance and response bias mathematically, avoiding arbitrary *a priori* algebraic corrections. The number of components was fixed to one to capture the variance shared across the memory measures. The resulting component was used to compute a composite memory score, termed MeCS. One-way ANOVA was used to compare groups in the MeCS.

MANCOVA was used to assess the main effects and interaction Group by CR levels on the brain structural covariance measures (expressed as GMC), covariating for age. Of note, Total Intracranial Volume (TIV) was not included as a covariate in the statistical models. Unlike traditional voxel-based morphometry, SBM utilizes ICA to extract multivariate patterns of spatial covariance across the whole brain. Because participant loadings reflect the relative expression or weight of distributed structural networks rather than absolute regional or global volumes, adjusting for TIV is redundant within this multivariate framework and risks over-correcting the data, a methodological approach fully consistent with established SBM validation and application protocols (Xu et al., 2009; Rashidi et al., 2026). ANCOVAs on single networks and relative Tukey’s post-hoc were also performed.

To investigate the independent and combined effects of brain structural networks and CR on cognitive performances, a centralized multiple linear regression model was performed on the entire sample (*N* = 209). MMSE and MeCS scores were entered as dependent variables in separate models. The regression model included Age, clinical group (entered as dummy variables), CR levels (expressed as z-scores), and brain measures (GMC of the identified structural networks) as independent predictors using the Enter method. Interaction terms (Group × CR and Group × GMC) were included in the models. All continuous predictors were mean-centered before the analysis to improve the interpretability of coefficients and reduce potential multicollinearity, which was formally assessed using the Variance Inflation Factor (VIF). For the centralized multiple linear regression model, the global model significance was set at *p*-level < 0.05, and individual predictors were evaluated at a nominal threshold of *p*-level < 0.05.

Subsequently, Pearson’s correlation analyses were performed to evaluate the direct strength and direction of the relationships between the GMC of the identified networks and cognitive scores (MMSE and MeCS), both in the entire sample and within each clinical group separately. To account for multiple comparisons, *p*-values were adjusted using Bonferroni’s correction (*p*-level 0.05/3 = 0.016).

Finally, to specifically test the conditional effect of CR on the relationship between brain atrophy and cognition, moderation analyses were performed. Moderation analyses were conducted to investigate whether the relationship between GMC of structural networks (*X*) and the MMSE scores and MeCS (*Y*) varied as a function of CR level (*M*). Age and sex were used as covariates of no interest. The analyses were performed using the PROCESS macro for SPSS-25 implemented in IBM SPSS Statistics, following the conditional process modeling framework. Specifically, moderation effects were tested using ordinary least squares regression models corresponding to PROCESS Model 1 (a single model was estimated). To strictly control for multiple comparisons across the 30 independent framework combinations (5 diagnostic strata [entire sample, AD, a-MCI, SCD and HS groups] × 3 brain networks × 2 cognitive outcomes), Bonferroni’s correction was applied to the omnibus global models (the omnibus *F*-test), setting the significance threshold at *α* = 0.05/30 *p*-level = 0.002. Within this globally protected variance space, for only those global models meeting this conservative threshold, the subsequent evaluation of specific interaction terms and conditional effects (simple slopes) was performed using a standard nominal threshold of *p*-level < 0.05 (uncorrected). This approach ensures strict family-wise error protection at the model level while preventing the excessive loss of statistical power (Type II error) that would result from applying a direct Bonferroni correction to individual interaction coefficients, which notoriously suffer from reduced statistical power in clinical neuroimaging samples where predictors share common variance.

This staged hierarchical approach, moving from centralized multiple regressions to correlation analyses and then to moderation models, was adopted to distinguish between the independent predictive value of GMC and CR, their associations, and the modulating role of CR across the AD continuum.

## 3 Results

### 3.1 Demographic and clinical characteristics

As shown in Table 1, there was a significant difference in mean age among groups (*F*_3,205_ = 14.6, *p* < 0.0001), with AD patients being older than HS (*p* = 0.005) and SCD (*p* < 0.00001) individuals. A-MCI patients were also older than SCD (*p* = 0.033) and HS (*p* < 0.0001) individuals.

**Table 1:** Demographic and clinical features.

Participant characteristics	AD	a-MCI	SCD	HS
	*N* = 57	*N* = 59	*N* = 44	*N* = 49
Age (mean ± SD)	73.2 ± 6.9^*°^	72.2 ± 8.2^#£^	68.2 ± 7.1	64.6 ± 8.1
Years of formal education (mean ± SD)	10.0 ± 4.4^*°§^	12.8 ± 4.5	13.0 ± 3.4	14.3 ± 3.9
Sex F/M	38/19	32/27	27/17	31/18
MMSE score (mean ± SD)	21.4 ± 4.0^*§°^	26.8 ± 2.0	27.5 ± 3.7	27.5 ± 4.4
MTA (mean ± SD)	2.4 ± 0.9^*§°^	1.9 ± 0.9^#£^	0.6 ± 0.8	0.7 ± 0.9
GMC of OTIN (mean ± SD)	−17978.4 ± 34809.1^*§°^	1522.9 ± 28920.7	8823.3 ± 27535.9	11149.6 ± 30007.7
GMC of PreCiN (mean ± SD)	−21790.0 ± 26716.4^*§°^	−1900.1 ± 34350.2^#£^	15446.6 ± 29642.0	12929.3 ± 26338.3
GMC of CHiN (mean ± SD)	−1822.9 ± 28212.8	−4177.2 ± 31866.3	4833.4 ± 32481.9	3405.5 ± 36218.9

*p < 0.05 AD vs. HS; ^#^*p* < 0.05 a-MCI vs. HS; ^§^*p* < 0.05 AD vs. a-MCI; °*p* < 0.05 AD vs. SCD; ^£^*p* < 0.05 a-MCI vs. SCD; ^*p* < 0.05 SCD vs. HS. AD, Alzheimer’s disease; a-MCI, amnestic mild cognitive impairment; SCD, subjective cognitive decline; HS, healthy subjects; MMSE, mini-mental state examination score; MTA, medial temporal lobe atrophy scale; GMC, grey matter concentration; OTIN, orbitofrontal-temporal-insular network; PreCiN, precuneus-cingulate network; CHiN, cingulate-hippocampal network.

In addition, there was a significant difference in years of formal education (*F*_3,205_ = 10.5, *p* < 0.0001) across groups. Post-hoc analyses revealed that AD patients were less educated than the other groups (AD vs. MCI and AD vs. SCD: *p* < 0.002; AD vs. HS: *p* < 0.0001). Sex distribution did not differ significantly among groups (Supplementary Table S1). Consistent with different AD stages, there were significant differences in MTA scores (*F*_3,205_ = 52.7, *p* < 0.0001) with AD and a-MCI patients showing greater atrophy than all other groups (*p* < 0.001 for all comparisons).

### 3.2 Analyses on neuropsychological measures

PCA conducted to extract the MeCS indicated that the first component (eigenvalue = 4.19) accounted for 70% of the total variance. This component exhibited robust factor loadings across all measures: W-IR = 0.909, W-DR = 0.932, SS-IR = 0.853, SS-DR = 0.899, W-HR = 0.717 and a negative loading of W-F = −0.671.

Significant differences among groups were observed in MMSE scores (*F*_3,205_ = 126.9, *p* < 0.0001) (violin plots of the distribution of MMSE scores across all diagnostic groups are reported in Supplementary Figure S21) and in the MeCS (*F*_3,205_ = 120.1, *p* < 0.0001), with AD and a-MCI patients exhibiting lower scores than the other groups (*p* < 0.0001 in each comparison). Between-group comparisons for all neuropsychological battery tests are provided in Supplementary Table S2.

### 3.3 Source-based morphometry

ICASSO revealed that all ICA components showed good convergence and stable decomposition (Iq > 0.95), indicating high reliability of the brain structural networks extracted.

**Figure 1:**
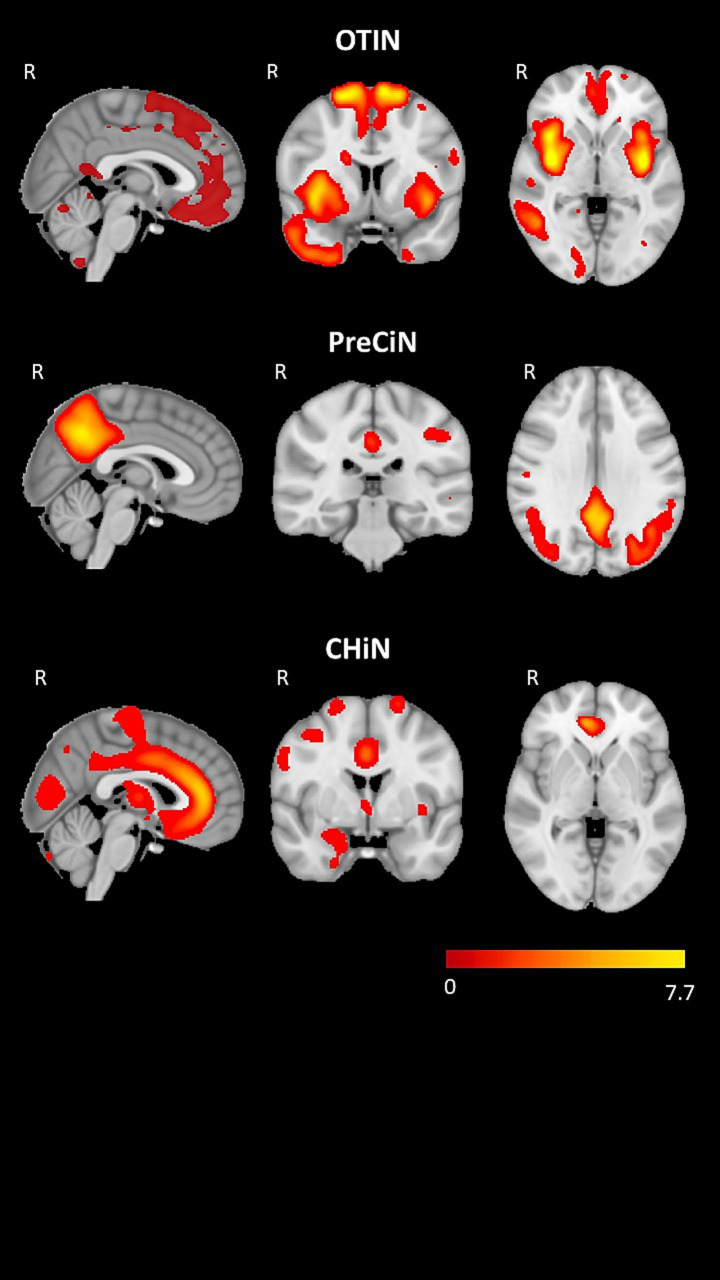
The three main components of grey matter covariance (GMC) identified by SBM analysis. The first component, OTIN (upper row), included orbitofrontal regions, the sub-genual region, and the temporal and insular cortices. The second component, PreCiN (middle row), included the precuneus and the posterior cingulate cortex. The third component, CHiN (lower row), included the entire cingulum, the orbitofrontal cortex, and the right hippocampus. The maps display the spatial distribution of structural covariance across grey matter regions within each network. These representations are provided for visualization purposes only and do not convey quantitative values. Network maps are overlaid on the MNI-152 template using the FMRIB Software Library (FSL; https://fsl.fmrib.ox.ac.uk/fsl/); R, right.

As shown in Figure 1, the two-step validation protocol in SBM analysis identified three main components of GMC. The component corresponding to the 6th component extracted including the GMC of orbitofrontal regions (BA10, 11, 12), sub-genual region (BA25), temporal (BA20, 21, 22, 41, 42), and insular (BA13, 14) cortices, was named the Orbitofrontal-Temporal-Insular Network (OTIN). The component corresponding to the 8th component extracted, including the GMC of the precuneus (BA7) and posterior cingulate cortex (BA31, 23), was named the Precuneus-Cingulate Network (PreCiN). Finally, the component corresponding to the 12th component extracted, including the GMC of the entire cingulum (anterior: BA32, 24; posterior: BA31, 23), orbitofrontal cortex (BA12), and the right hippocampus, was named the Cingulate-Hippocampal Network (CHiN). In Table 1 are mean ± SD of the loadings of the GMC of the OTIN, PreCiN, and CHiN.

**Figure 2:**
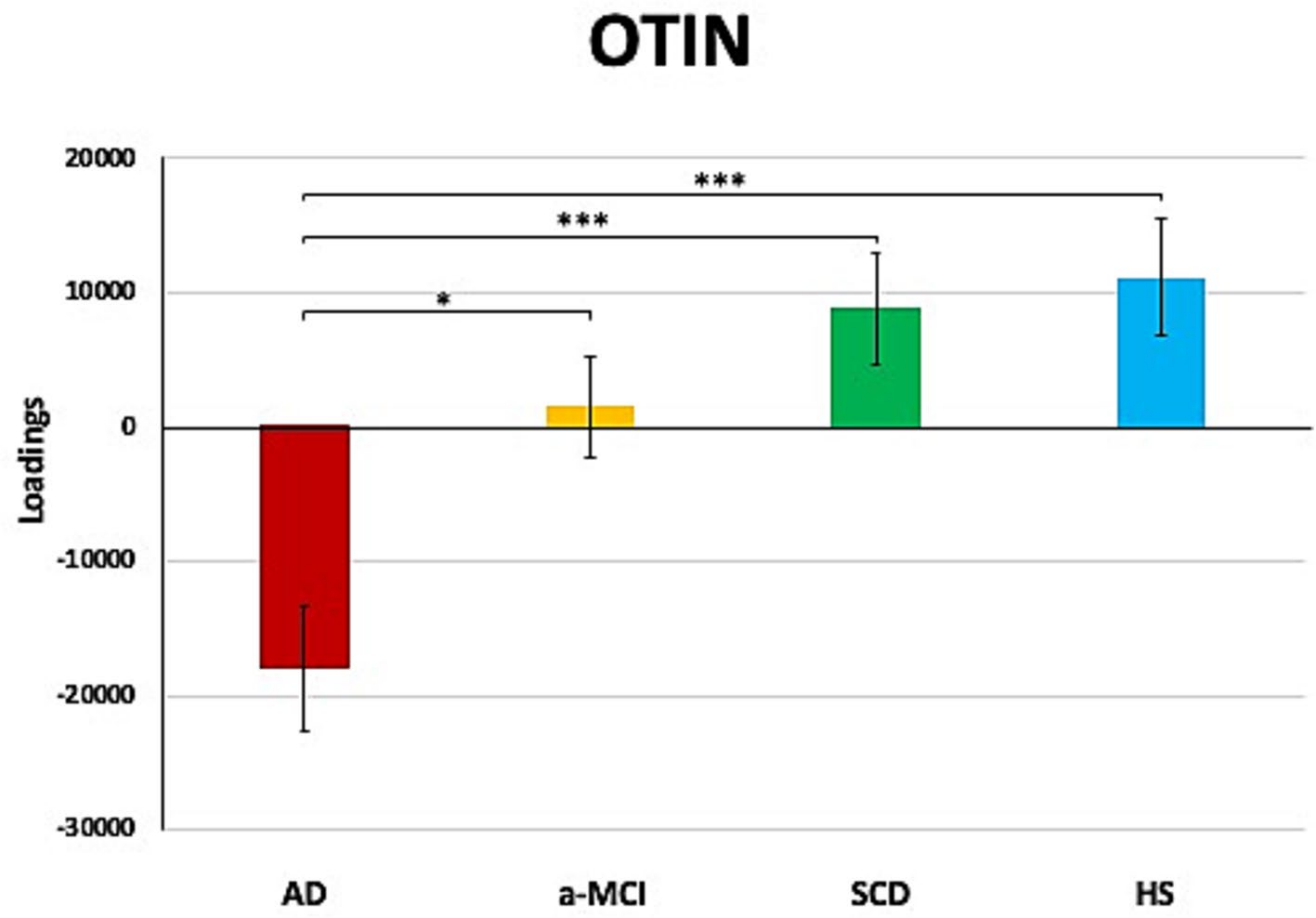
Group differences in grey matter covariance within the Orbitofrontal-Temporal-Insular Network (OTIN). Significant effects were observed (*p ≤ 0.05; ***p ≤ 0.0001), reflecting lower grey matter covariance in the Alzheimer’s disease (AD) group compared with the amnestic mild cognitive impairment (A-MCI), subjective cognitive decline (SCD), and healthy subjects (HS) groups.

**Figure 3:**
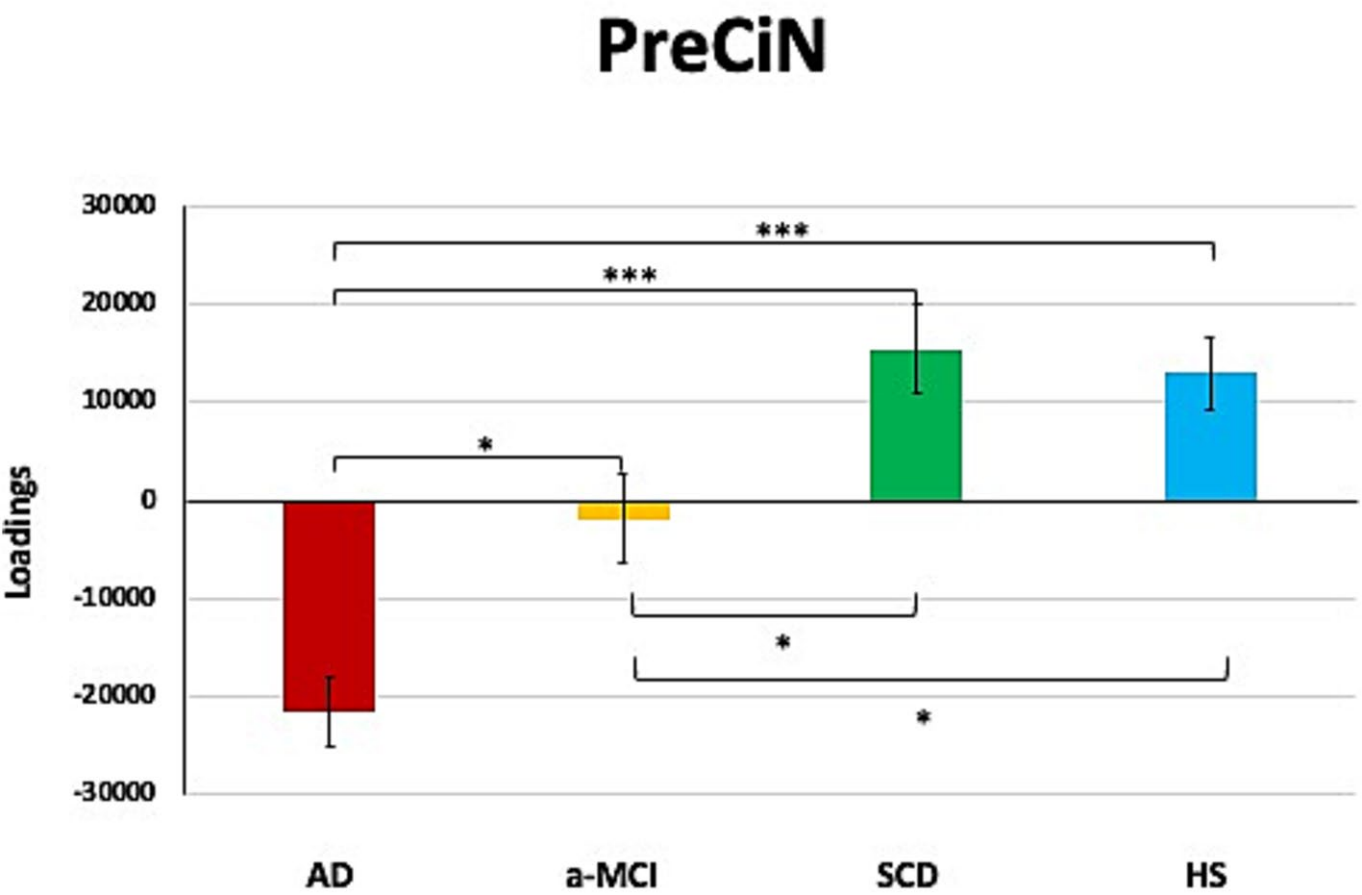
Group differences in grey matter covariance within the Precuneus-Cingulate Network (PreCiN). Significant effects were detected (*p ≤ 0.05; ***p ≤ 0.0001), indicating reduced covariance in the AD group relative to the a-MCI, SCD, and HS groups. In addition, a significant covariance reduction (*p ≤ 0.05) was observed in the a-MCI group compared to the SCD and HS groups.

**Figure 4:**
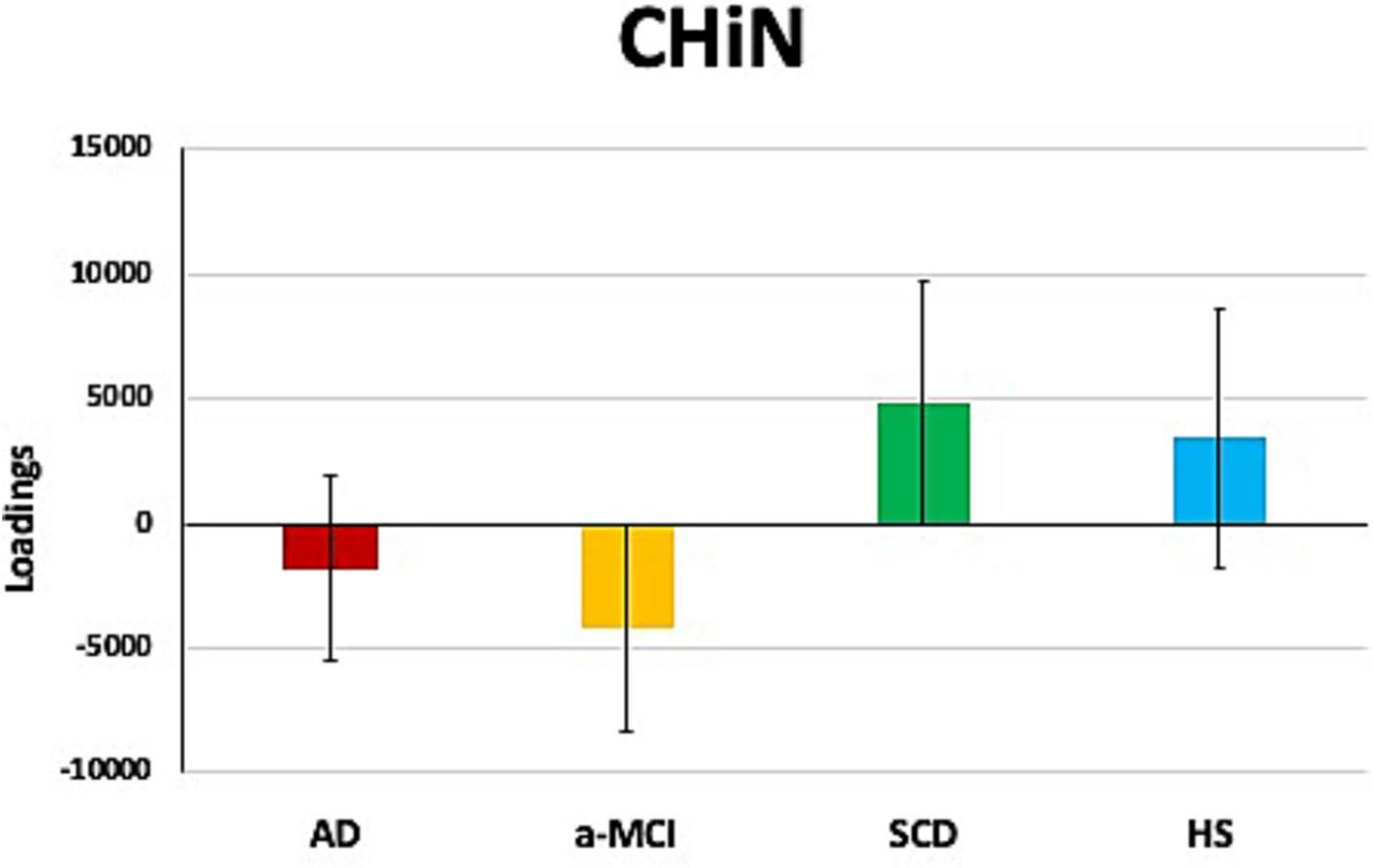
Group-specific grey matter covariance in the Cingulate-Hippocampal Network (CHiN). No significant group effects were found.

A MANCOVA on the GMC of the three cortical networks showed a significant main effect of Group (Pillai trace *V* = 0.233, *F*_9,603_ = 5.65, *p* < 0.0001). Specifically, the ANOVAs showed: (i) a significant main effect of Group in the covariation of GMC in the OTIN (*F*_3,201_ = 9.85, *p* < 0.0001) (Figure 2 and Table 1). *Post hoc* analyses showed a significant reduction in OTIN GMC in AD compared with a-MCI patients (*p* = 0.004; CI 95%: −34230.9; −4771.6), SCD (*p* < 0.0001; CI 95%: −42717.4; −10886.0), and HS (*p* < 0.0001; CI 95%: −44578.6; −13677.4) groups. No other significant results were observed in *post hoc* analyses; (ii) a significant main effect of Group in the covariation of GMC in the PreCiN (*F*_3,201_ = 17.38, *p* < 0.0001) (Figure 3 and Table 1). Post hoc analyses showed significant GMC reduction in AD patients compared with a-MCI (*p* = 0.002; CI 95%: −34124.4; −5655.4), SCD (*p* < 0.0001; CI 95%: −52617.3; −21856.0), and HS (*p* < 0.0001; CI 95%: −49650.5; −19788.0) groups; a significant GMC reduction was observed in the PreCiN of a-MCI patients compared with SCD (*p* < 0.019; CI 95%: −32613.4; −2080.1) and HS (*p* = 0.05; CI 95%: −29643.1; −15.60) groups. (iii) In the CHiN, no significant main effect of Group was observed (*F*_3,201_ = 0.91, *p* = 0.432) (Figure 4 and Table 1); (iv) no significant main effect of CR level (Pillai trace *V* = 0.013, *F*_3,199_ = 0.888, *p* = 0.448) was found; (v) no significant interaction between Group and CR level (Pillai trace *V* = 0.043, *F*_9,603_ = 0.888, *p* = 0.468) was found.

### 3.4 Regression analyses

The VIF values range from [1.6] to [8.1] indicating the absence of severe multicollinearity issues among most of the independent variables. In the entire sample, MMSE scores were significantly predicted by the global regression model (*R*_adj_^2^ = 0.702, *F*_20,188_ = 20.6, *p* < 0.0001), and by single predictors, such as Age (*p* = 0.045) and Group (AD and a-MCI *p* < 0.0001, respectively), while the MeCS was significantly predicted by the global regression model (*R*_adj_^2^ = 0.777, *F*_20,188_ = 24.3, *p* < 0.0001) and by single predictors as Age (*p* < 0.0001) and Group (AD and a-MCI *p* < 0.0001, respectively) and by interaction terms CR × AD (*p* = 0.040) and CR × a-MCI (*p* = 0.024).

All statistical details of the regression models are reported in Supplementary Table S3.

### 3.5 Inter-network correlations

Significant correlations between the GMC of OTIN and PreCiN were found in the entire sample (*r* = 0.57, *p* < 0.0001). Notably, the association between the GMC of OTIN and PreCiN was not merely a reflective property of the total sample; rather this relationship remained robust and statistically significant within each single diagnostic group (AD: *r* = 0.41, *p* = 0.002; a-MCI: *r* = 0.52, *p* < 0.0001; SCD: *r* = 0.66, *p* < 0.0001; HS: *r* = 0.43, *p* = 0.002) confirming that these network interactions persists across the entire clinical spectrum. Conversely, no significant correlations between the GMC of CHiN and the entire sample or the single groups were observed (Supplementary Table S4).

### 3.6 Correlations between structural networks, CR level, or cognitive performances

CR level showed a significant association with MeCS only in the HS group (*r* = 0.43, *p* = 0.007); no other significant correlations were found. In the entire sample, we found significant correlations between the GMC of OTIN and PreCiN with MMSE (OTIN: *r* = 0.31, *p* < 0.0001; PreCiN: *r* = 0.42, *p* < 0.0001) and MeCS (OTIN: *r* = 0.40, *p* < 0.0001; PreCiN: *r* = 0.42, *p* < 0.0001) and between the GMC of CHiN and MeCS (*r* = 0.22, *p* = 0.006). In the single groups, no significant correlations were found (Supplementary Table S5).

### 3.7 Moderation analyses

#### 3.7.1 General cognitive efficiency

In the entire sample, significant global effects were found between MMSE scores, GMC of OTIN, PreCiN and CHiN, and CR level: (i) model including *X* = OTIN, *Y* = MMSE and *M* = CR level: *R* = 0.44, *F*_5,203_ = 9.30, *p* < 0.0001; (ii) model including *X* = PreCiN, *Y* = MMSE and *M* = CR Level: *R* = 0.49, *F*_5,203_ = 12.44, *p* < 0.0001; (iii) model including *X* = CHiN, *Y* = MMSE and *M* = CR Level: *R* = 0.40, *F*_5,203_ = 7.43, *p* < 0.0001. Table 2 reports the beta values of the predictors and the conditional effects of significant interactions.

**Table 2:** Moderation analyses for general cognitive efficiency (MMSE score).

Group	Effects of the model			
	Predictors	Beta coefficient	*t*	*p*-value
Entire sample	OTIN	0.01	3.18	0.002
	CR level	0.18	0.77	0.440
	OTIN × CR level	0.01	0.86	0.391
	PreCiN	0.01	4.89	<0.0001
	CR level	0.25	1.08	0.281
	PreCiN × CR level	0.01	−1.29	0.198
	CHiN	0.01	−0.96	0.334
	CR level	0.19	0.78	0.434
	CHiN × CR level	0.01	−1.29	0.1973

In the single groups, no significant effects were found (Supplementary Table S6).

#### 3.7.2 Memory performances

In the entire sample, significant main effects were found between MeCS, GMC of OTIN, PreCiN and CHiN, and CR level: (i) model including *X* = OTIN, *Y* = MeCS and *M* = CR Level: *R* = 0.59, *F*_5,203_ = 16.74, *p* < 0.0001; (ii) model including *X* = PreCiN, *Y* = MeCS and *M* = CR Level: *R* = 0.49, *F*_5,203_ = 15.19, *p* < 0.0001; (iii) model including *X* = CHiN, *Y* = MeCS and *M* = CR Level: *R* = 0.51, *F*_5,203_ = 11.10, *p* < 0.0001.

In AD patients, we did not observe significant main effects in any moderation analyses (Supplementary Table S7).

In a-MCI patients, significant main and interaction effects were found in the model, including *X* = OTIN, *Y* = MeCS, and *M* = CR level, *R* = 0.60, *F*_5,53_ = 4.77, *p* < 0.001; Interaction OTIN × CR Level: *R*^2^chng = 0.07, Cohen’s ƒ^2^ = 0.009, partial *η*^2^ = 0.086, *F*_1,53_ = 4.98, *p*_unc._ = 0.031. The conditional effects of interaction OTIN × CR are reported in Table 3. Remarkably, the moderation effect was significant for the higher CR levels only (level of moderator expressed as *z*-scores: 0.93; *t* = 2.02; *p*_unc._ = 0.049). No other significant moderation effect was found (Supplementary Table S7).

**Table 3:** Moderation analyses for memory performance (MeCS).

Group	Effects of the model					Conditional effects of *X* at different levels of the moderator			
	Predictors	Beta coefficient	*t*	*p*-value	Interaction	Levels of moderator	Effect	*t*	*p*-value
Entire sample	OTIN	0.01	5.50	<0.0001					
	CR level	−0.08	−1.30	0.194					
	OTIN × CR level	0.01	0.71	0.474					
	PreCiN	0.01	4.25	0.001					
	CR level	−0.05	−0.74	0.460					
	PreCiN × CR level	0.01	1.42	0.156					
	CHiN	0.01	0.29	0.766					
	CR level	−0.07	−1.07	0.282					
	CHiN × CR level	0.01	0.46	0.644					
a-MCI	OTIN	0.11	0.40	0.688	OTIN × CR level	−1.09	0.01	−1.00	0.321
	CR level	−0.18	−1.88	0.066		0.03	0.01	0.46	0.642
	OTIN × CR level	0.01	2.23	0.031		0.93	0.01	2.02	0.049
SCD	OTIN	0.01	−1.04	0.306					
	CR level	−0.01	−0.13	0.892					
	OTIN × CR level	0.01	1.31	0.199					
HS	OTIN	0.01	−0.07	0.942					
	CR level	0.09	1.53	0.135					
	OTIN × CR level	0.01	−0.71	0.480					
	PreCiN	0.01	−0.06	0.946					
	CR level	0.12	2.07	0.046					
	PreCiN × CR level	0.01	−0.66	0.512					
	CHiN	0.01	−0.36	0.721					
	CR level	0.13	2.19	0.035					
	CHiN × CR level	0.01	−1.40	0.170					

SCD individuals showed a significant global effect in the model, including *X* = OTIN, *Y* = MeCS, and *M* = CR Level: *R* = 0.66, *F*_5,38_ = 4.89, *p* < 0.002. No other significant effect (main effect of predictors or interaction) was found (Supplementary Table S7).

In the HS group, significant global effects were found in all analyses performed: (i) model including *X* = OTIN, *Y* = MeCS and *M* = CR Level: *R* = 0.74, *F*_5,43_ = 8.22, *p* < 0.0001; (ii) model including *X* = PreCiN, *Y* = MeCS and *M* = CR Level: *R* = 0.75, *F*_5,43_ = 8.20, *p* < 0.0001; (iii) model including *X* = CHiN, *Y* = MeCS and *M* = CR Level: *R* = 0.76, *F*_5,43_ = 8.91, *p* < 0.0001. No significant interactions were found. Specifically, in the HS group, although all global models for OTIN, PreCiN, and CHiN reached high statistical significance (as reported above), these effects were not driven by the structural integrity of the networks or by a moderation effect, as no significant interactions were found. The only exception for these findings was a weak but significant main effect of CR level, which independently predicted MeCS performance in the PreCiN model (*p*_unc._ = 0.046) and in the CHiN model (*p*_unc._ = 0.035) (Table 3).

Table 3 reports the beta values of the predictors and the conditional effects of significant interactions.

## 4 Discussion

The present study investigated how CR modulates relationships between cognitive performance and brain structural integrity across the continuum from cognitively healthy older adults to patients with dementia. Previous research suggested that CR plays an effective modulating role in specific time windows (Serra et al., 2017). The present findings hypothesize that CR is a stage-dependent mechanism that may exert its protective and compensatory efficacy, varying with the integrity of underlying neural substrates and disease stage. In interpreting these results, it is essential to consider the concepts of Brain Reserve (BR) and Cognitive Reserve (CR). In the present study, the relative structural covariance of the identified networks (GMC) can be viewed as an indicator of BR (Stern, 2012). In contrast, the moderation effects we observed are property-aligned with the role of CR, which theoretically represents the changing ability to maintain cognitive function despite relative structural reduction, a phenomenon widely attributed in the literature to underlying mechanisms of brain plasticity and compensatory strategies (Stern, 2012). Our results highlight the interplay between these two reserves. Specifically, we observed that high CR levels appear to mitigate or “decouple” the relationship between BR loss and clinical symptoms: at the same level of relative structural covariance attenuation of the network (considered as a potential indicator of reduced BR), individuals with higher CR demonstrated superior memory performance compared to those with lower CR. This suggests that while the GMC loadings reflect the relative neuroanatomical status of the disease, the CR might act as a stage-dependent modulator that could determine how much structural damage an individual can tolerate before manifest cognitive decline occurs.

A large cohort of participants across the AD spectrum was recruited in the current study. All participants exhibited the expected cognitive profile and clinical features. Specifically, we observed the expected pattern of medial temporal lobe atrophy (as indicated by the MTA scores), with a progressive increase from HS through SCD and a-MCI to AD patients.

To investigate the relationships among CR level, cognitive functioning, and brain structural integrity, an SBM analysis was used to identify patterns of GM covariation across brain regions critically involved in memory and executive functions. Three distinct networks involving the temporal lobes, precuneus, cingulate gyrus, and dorsal and orbitofrontal prefrontal cortices were selected to analyze their vulnerability across the AD continuum. Specifically, OTIN, encompassing orbitofrontal regions (BA10, 11, 12), sub-genual areas (BA25), temporal (BA20, 21, 22, 41, 42), and insular cortices (BA13, 14), showed progressive accumulation of atrophy across groups, with a significant reduction in AD patients compared to all other groups. PreCiN, comprising the precuneus (BA7) and posterior cingulate cortex (BA31, 23), showed an even more pronounced pattern of vulnerability, with detectable GMC reduction already evident at the early stage of a-MCI. In contrast to the other identified networks, the CHiN, despite including the right hippocampus, showed no significant between-group differences in GM concentration. This dissociation between macroscopic atrophy (clinically assessed via the MTA scale) and the stability of network covariance patterns (as revealed by SBM) suggests that the CHiN may represent a preserved, core neural substrate that consistently supports cognitive performance despite emerging pathology, reflecting what Stern and colleagues (2012, 2020) describe as the recruitment of alternative/efficient neural networks of the brain. The inclusion of sensory-motor and visual regions, areas typically spared in the early stages of the AD spectrum, further supports the hypothesis that this component captures a pattern of structural integrity rather than pathological volume loss. From a methodological standpoint, the multivariate nature of SBM may prioritize networks with high internal coherence across the entire sample, effectively identifying clusters of regions that maintain stable volumetric relationships regardless of the clinical group. Therefore, the CHiN likely reflects a structural organization that may remain relatively preserved, providing a complementary perspective to the localized, atrophy-driven changes observed in the OTIN and PreCiN networks.

However, the statistical properties of multivariate decomposition (as estimated by the SBM technique) might prioritize networks with stronger inter-correlations, and potentially obscure between-group differences that could be evident in region-of-interest-based analyses.

The distinct vulnerability of OTIN and PreCiN aligns with known patterns of AD neuropathology (Braak and Braak, 1991; Jack et al., 2013; Braak and Del Tredici, 2015). The precuneus and posterior cingulate cortex, key hubs of the default mode network (Raichle and Snyder, 2007; Buckner et al., 2008; Andrews-Hanna et al., 2010; Foster and Koslov, 2025), are among the earliest sites of amyloid deposition (Klunk et al., 2004; Sperling et al., 2009; Palmqvist et al., 2017) and hypometabolism even at preclinical stages of AD (Minoshima et al., 1997; Mosconi et al., 2010). Similarly, orbitofrontal and temporal-insular regions are vulnerable to early AD pathology and play a critical role in memory, emotional regulation, and interoceptive processing (Thal et al., 2002; Gupta et al., 2019; Serra et al., 2023). Notably, OTIN and PreCiN structural integrity showed high intercorrelation across all groups, suggesting shared vulnerability or coordinated degeneration. This strong coupling between networks may reflect their joint participation in large-scale brain systems, such as the default mode and salience networks, since early AD stages (Seeley et al., 2007; Ribeiro da Costa et al., 2022). Across the entire sample, the regression analyses revealed that general cognitive performance, as measured by MMSE scores, was significantly predicted by the global model. Within this model, PreCiN structural integrity emerged as a robust predictor, highlighting its central role in supporting cognitive functioning regardless of disease stages (Buckner et al., 2005; Koch et al., 2022). OTIN, although not reaching statistical significance in the Bonferroni-corrected global model, displayed a nominal association with MMSE scores. Given its well-established involvement in memory, emotional regulation, and interoceptive processes (Craig, 2009; Rolls et al., 2020; Seeley et al., 2007), OTIN should still be considered a critical network contributing to cognitive outcomes. Notably, the diagnostic group accounted for the largest proportion of variance, emphasizing the interplay between disease stage and network integrity. For memory performance, as measured by MeCS, the global regression model was similarly significant across the sample, with Age and Group emerging as key predictors. Again, OTIN, while not statistically significant under strict correction, may play a supportive and biologically meaningful role in memory processes. These findings suggest that the structural integrity of both PreCiN and OTIN provides a fundamental neural scaffold for cognition, with PreCiN showing clear predictive validity and OTIN representing a network of theoretical and functional importance.

Regarding CR, while it appears to exert protective effects in healthy aging, its modulatory influence on the relationship between brain structure and cognitive performance seems to change in the presence of AD pathology. This modulatory effect is suggested to decline during the transition from SCD to a-MCI and potentially loses effectiveness at more advanced AD stages. Specifically, in cognitively healthy individuals, CR may represent the current efficient cognitive and neural resources accumulated over the lifespan. Importantly, we propose that CR is not a fixed resource that stops accumulating after early adulthood; rather, it can continue to increase even in late life through ongoing cognitive engagement, learning new skills, and maintaining intellectually stimulating activities (Serra et al., 2024). This ongoing accumulation is thought to support the maintenance of cognitive performance in healthy aging (Stern, 2009; Barulli and Stern, 2013; Cabeza et al., 2018). Conversely, in AD patients, extensive neuropathology might overwhelm the hypothesized compensatory capacity of the brain, making CR no longer effective in compensating for cognitive deterioration (Serra et al., 2017). Critically, we propose that, in the early stages of AD, such as a-MCI and even in SCD conditions, CR may interact with brain areas that are relatively spared by tissue damage, contributing to a potential stage-dependent modulation of cognitive performance. Ultimately, the present results suggest that the relationship between CR and cognitive performance varies across disease stages. In HS, CR emerged as a significant predictor of memory performance, consistent with the traditional view of reserve as a protective factor accumulated across the lifespan (Stern, 2012; Pettigrew and Soldan, 2019; Varela-López et al., 2022). The moderation analyses showed that, in HS, the global models reached statistical significance in the analyses involving all networks (OTIN, PreCiN, and CHiN). However, a detailed inspection of the predictors revealed that these models were not driven by an interaction effect, as no significant moderations were found. Critically, this pattern differs across stages of the AD continuum. In the HS group, network integrity did not independently predict memory, and the global significance was supported only by a weak but significant main effect of CR level in the PreCiN and CHiN models. In the SCD group, CR no longer correlated with memory performance, and moderation analyses revealed only a global effect of OTIN, with no significant main effect of conditions or interactions. This transition suggests that even in the earliest preclinical stages of cognitive decline, when objective cognitive deficits are not yet detectable on formal testing, the mechanisms by which CR operates can begin to change. The fact that CR and structural integrity act primarily as independent predictors (or show no effect) in HS and SCD individuals, rather than through a formal moderation, suggests that compensatory mechanisms are not yet active or follow additive rather than interactive pathways in these conditions. The presence of subjective cognitive complaints, despite a normal neuropsychological performance, may reflect subtle alterations in the efficiency of compensatory mechanisms rather than gross structural loss (Serra et al., 2026). Individuals with SCD may experience greater cognitive effort or increased fatigability to maintain normal performance levels. These phenomenological aspects, which cannot be captured by standard cognitive measures, are internally perceived by individuals themselves.

The most prominent indication for stage-dependent CR effects emerged in the a-MCI group. We observed a significant, albeit slight, interaction between OTIN structural integrity and CR level in predicting memory performance. This interaction suggests that at this transitional stage, where neuropathological burden is increasing but may not yet have fully overwhelmed the brain’s compensatory capacity, CR potentially moderates the relationship between brain structure and cognitive function. Individuals with higher CR appear better able to leverage residual OTIN structural integrity to maintain memory performance, which is compatible with the engagement of compensatory neural strategies in response to emerging pathology. These findings provide supportive evidence for the previously hypothesized notion that a-MCI represents a critical window in which reserve mechanisms might be actively recruited to mitigate the impact of accumulating neuropathology (Serra et al., 2017).

In stark contrast, in AD patients, neither CR nor its interaction with structural networks significantly predicted cognitive performance. In the regression models, MMSE scores were not predicted by any variable, while memory performance showed only a weak relationship with OTIN integrity. The moderation analyses did not reveal any significant effects. At this stage, widespread neuronal loss, synaptic dysfunction, and network disruption are hypothesized to make previously effective compensatory mechanisms ineffective. This observation leads us to hypothesize the presence of a ‘tipping point’ in neurodegeneration (Jack et al., 2013; Jack et al., 2018), while not directly tested in the current cross-sectional design, this framework provides a highly consistent hypothesis according to which, beyond such a critical threshold, cognitive reserve might no longer mitigate the clinical expression of pathology. The overwhelmed capacity of remaining neural resources to support cognitive function could render previously protective factors (such as educational attainment) insufficient to maintain performance in AD patients.

The present findings support a revised conceptual model of CR that emphasizes its stage-dependent nature. Rather than viewing CR as a fixed quantity that uniformly protects against cognitive decline, our model recognizes that reserve may operate through distinct mechanisms at different disease stages: phenomena theoretically associated with neural efficiency in healthy aging (shown by direct CR-cognition correlations and global moderation effects), and cognitive compensation in a-MCI stages (shown by specific CR × brain structure interactions), leading ultimately to an ineffective compensation at the AD stage (shown by the absence of any CR modulation). This reconceptualization has potential implications for how we measure, interpret, and potentially enhance CR. Thus, interventions aimed at enhancing reserve, whether through cognitive training, physical activity, or social engagement, might be particularly effective before extensive neuropathology has accumulated. The a-MCI stage appears to be a potential critical time window (Serra et al., 2017) in which reserve mechanisms might be actively engaged but not yet overwhelmed, thus suggesting that a-MCI patients could represent a highly suitable target for reserve-enhancing interventions. The SCD stage, while initially showing potentially disrupted CR mechanisms, may represent an even earlier window where interventions could help to prevent or delay the transition to overt cognitive impairment.

A key finding of our study is the differential sensitivity of cognitive measures in moderation analyses. While the MMSE, a global screening tool, only showed significant global effects in the pooled sample, the MeCS (the memory composite Score) revealed significant global effects even within the single groups, such as a-MCI, SCD, and HS. Remarkably, the emergence of a significant interaction between OTIN and CR Level, which was observed only in the a-MCI group highlights that the potential modulating role of CR is stage-dependent and may be more easily captured by refined neuropsychological composite measures. The fact that these interaction effects disappear when using the MMSE, but persist with the MeCS at the group level, suggests that the lack of significance reported in some models is not merely a matter of sample size but might also depend on the granularity of the clinical outcome. While the whole-sample analysis provides maximum statistical power, the group-specific results for MeCS suggest that our findings may reflect processes that are already active in the early phases of the AD continuum.

Some limitations should be considered when interpreting these findings. The CR was operationalized solely by educational attainment. Although education is a well-established proxy for CR (Stern, 2012; Arenaza-Urquijo and Vemuri, 2018; Pettigrew and Soldan, 2019), it captures only one component of lifelong cognitive enrichment that also encompasses occupational complexity, leisure activities, and social engagement across the lifespan (Stern et al., 2020; Pappalettera et al., 2024). Importantly, in the present study, years of formal education differed significantly between groups, with AD and a-MCI patients showing lower educational attainment compared to SCD individuals and HS. This between-group difference in the CR proxy itself warrants careful consideration when interpreting stage-dependent CR effects. Specifically, the lower educational attainment observed in AD patients may partially reflect cohort effects, selective survival, or disease-related factors rather than purely reserve-related processes. Moreover, floor effects in both education and cognitive performance in the AD group cannot be entirely excluded as contributing factors to the absence of moderation effects at this stage. However, several methodological choices were made to mitigate these concerns. Age was included as a covariate in all analyses, including the MANCOVA, the multiple regression models, and the moderation analyses, where age and sex were entered as covariates of no interest. CR level was entered as a continuous mean-centered *z*-score variable in all models, preserving individual variability within each group and reducing the risk that group-level differences in mean education drive the observed effects. Furthermore, the stage-dependent pattern of CR effects does not follow a simple monotonic gradient parallel to the educational attainment differences between groups: critically, a significant CR × OTIN interaction emerged only in a-MCI patients, who are less educated than SCD individuals where no moderation was found, arguing against a straightforward demographic confounding explanation. Future studies should incorporate composite, multidimensional measures of CR, including occupational complexity, leisure activities, and social engagement, as assessed for instance through validated tools such as the Global Enrichment Index (Serra et al., 2024, 2026), and should employ larger, demographically balanced samples across disease stages to better disentangle the contributions of educational attainment and disease-related factors to the observed CR effects.

Furthermore, the cross-sectional design precludes definitive conclusions about the temporal changes in reserve mechanisms. Longitudinal studies tracking individuals across disease stages are needed to directly test whether the stage-specific mechanisms inferred from our cross-sectional design are confirmed. Importantly, our sample included individuals with biomarker-confirmed AD pathology (in the MCI and AD groups), diagnosed according to established research criteria (Jack et al., 2024; Albert et al., 2011), whereas the SCD and healthy control groups were defined based on clinical and neuropsychological criteria without biomarker confirmation. While this approach facilitates clinical identification of the groups, this asymmetry introduces uncertainty regarding the precise positioning of all participants along the same pathological continuum, as the presence of undetected preclinical pathology cannot be excluded. This is particularly relevant for the SCD group, which is known to be a heterogeneous condition, with a subset of individuals likely harboring early amyloid or tau pathology despite the absence of objective cognitive deficits on formal testing (Jessen et al., 2014, 2020), meaning some cases are likely to progress to an overt cognitive impairment, while others do not. The absence of biomarker stratification in these groups may therefore introduce noise in the stage-dependent CR effects observed, particularly in the SCD group where only limited and non-significant CR effects emerged. However, it is noteworthy that several clinical indicators support the validity of the HS classification in the present sample: all HS participants were free of subjective cognitive complaints, performed within normal limits on all neuropsychological measures, and showed no clinically relevant signs of atrophy of the medial temporal lobe and hippocampal structures (as measured by the MTA scale), permits reasonably categorizing these individuals as healthy subjects. These converging criteria reduce, albeit do not eliminate, the probability of undetected preclinical pathology in this group. It is recommended that future studies incorporating biomarker-based stratification, especially in SCD and healthy aging populations, as well as longitudinal follow-up, be conducted. This would allow a more precise characterization of the relationship between reserve, pathology, and cognitive trajectories.

Again, although participants underwent rigorous clinical screening to be included in this study (including exclusion of individuals with major systemic or neurological diseases, psychiatric disorders, and Hachinski Ischemic Score >4, to minimize overt vascular and psychiatric confounders), the study did not include quantitative measures of metabolic, cardiovascular, or subclinical psychological factors. In particular, future studies should include quantitative measures of body mass index (BMI), cardiovascular risk factors, and subclinical psychological symptoms such as anxiety and depression. These variables may independently influence grey matter integrity and subjective cognitive complaints, and their absence represents a limitation that future studies should address by incorporating standardized assessments of these dimensions alongside neuroimaging and cognitive measures. Finally, it should be noted that while the inter-network correlations (e.g., between OTIN and PreCiN) were robust across all clinical groups, the associations between these networks and cognitive measures (MMSE and MeCS) reached statistical significance only when analyzing the entire sample, disappearing when groups were analyzed individually. This could be considered a limitation, as the reduced sample size of single diagnostic groups may have limited the statistical power to detect stage-specific clinico-anatomical relationships. Furthermore, the lack of significant correlations within single groups might be attributed to a restriction of range effect; the relatively narrow variance in both GM integrity and cognitive performance within a single clinical stage can mask associations that only emerge when considering the full spectrum of AD progression. Nevertheless, the presence of a stage-specific moderation effect exclusively in the a-MCI group suggests that while simple linear brain-cognition correlations require full across-spectrum variance to reach significance, the CR operates through distinct, non-linear mechanisms that are strictly dependent on the clinical stage.

Future studies with larger, balanced groups are needed to confirm whether these relationships also exist independently within specific diagnostic categories.

In conclusion, the present study provides supportive evidence that CR operates as a stage-dependent mechanism across the AD continuum. While CR appears to exert protective effects in healthy aging, a phenomenon conceptually linked to enhanced neural efficiency, its role is hypothesized to shift toward compensation in the a-MCI stage, where higher reserve may enable more effective use of residual structural brain resources. In the SCD condition, the relationship between CR and cognition is subtly altered, suggesting potential changes in neural efficiency despite preserved cognitive performance. Ultimately, when neuropathological burden becomes extensive in advanced AD, reserve mechanisms appear to be overwhelmed and may lose their compensatory effectiveness.

This lack of a significant individual moderation observed in HS and SCD groups compared to the significant interaction found in the a-MCI group suggests that CR may act as a potential “additive bonus” in cognitively unimpaired individuals but could transform into an “active buffer” (reflecting a specific moderation effect) only when neurodegeneration is hypothesized to reach a clinically relevant threshold, such as in a-MCI patients.

The present study underscores the importance of considering disease stage when evaluating the effects on reserve and suggests that early stages may represent critical windows for interventions aimed at enhancing or preserving cognitive reserve. The identification of distinct structural networks exhibiting differential vulnerability across disease stages and their varying relationships with CR and cognitive performance provides important insights into the potential neural substrates supporting cognitive function and theories of compensation across the AD continuum.

## Data Availability

The raw data supporting the conclusions of this article will be made available by the authors, without undue reservation.

## Footnotes

1
https://trendscenter.org/software/gift/

2
https://www.ibm.com/it-it/analytics/spss-statistics-software
