# Supplementary material for: Stage-dependent effects of cognitive reserve on memory and brain structural integrity across the spectrum from healthy aging to Alzheimer’s disease
Source: Front Hum Neurosci. 2026 Jul 15;20:1830181. doi: 10.3389/fnhum.2026.1830181 (PMC13415583; doi:10.3389/fnhum.2026.1830181)
Supplement: Supplementary file 1 [file Data_Sheet_1.pdf]

Supplementary Materials

Demographical results

As reported in the manuscript and shown in Table S1, sex distribution did not differ significantly among groups:

Table S1. Sex distribution

Groups	χ^2 (df=1)	p-level
AD *vs.* a-MCI	1.87	0.171
AD *vs.* SCD	0.30	0.580
AD *vs.* HS	0.13	0.710
a-MCI *vs.* SCD	0.52	0.470
a-MCI *vs.* HS	0.90	0.340
SCD *vs.* HS	0.04	0.850

Results obtained by participants at neuropsychological battery

Table S2 reports the results obtained by groups at neuropsychological battery.

Specifically, significant group differences emerged across all cognitive domains (all ps < 0.001). More in detail, in verbal episodic memory, performance differed markedly among groups. AD patients showed the lowest scores on the 15-Word List immediate recall (26.6 ± 7.9), delayed recall (2.0 ± 2.9), recognition hits (9.1 ± 3.7), and the highest false recognitions (9.5 ± 6.8), followed by a-MCI, SCD, and healthy subjects (HS), who consistently showed the best performance (all group effects: $Fs_{3,209}$ = 33.1–96.2, ps < 0.001). A similar gradient was observed for Short Story recall, with AD patients performing worst and HS best for both immediate and delayed

recall ($Fs_{3,209}$ = 45.5–57.9, ps < 0.001). For visuo-spatial episodic memory, Rey's Complex Figure immediate and delayed recall also revealed significant group effects ($Fs_{3,209}$ = 37.5–38.6, ps < 0.001), with AD patients performing worst, followed by a-MCI, while SCD and HS showed comparable and higher scores. Short-term memory measures showed smaller but significant group effects. Digit Span forward and backward differed among groups ($Fs_{3,209}$ = 4.9 and 9.4, respectively, ps < 0.001), with lower performance in AD patients compared to the other groups. Similarly, visuo-spatial span (Corsi forward and backward) showed significant group differences ($Fs_{3,209}$ = 16.4 and 21.0, ps < 0.001), with AD patients performing worst and HS best. Executive functions were also significantly different across groups. Phonological verbal fluency and the number of criteria achieved on the Modified Card Sorting Test showed strong group effects ($Fs_{3,209}$ = 19.1 and 40.2, respectively, ps < 0.001), with progressively better performance from AD to HS. Reasoning ability, assessed with Raven's Progressive Matrices, differed significantly between groups ($F_{3,209}$ = 25.2, p < 0.001), again showing the lowest scores in AD and the highest in HS. Finally, language and visuoconstructional abilities also showed significant group differences. Naming performance (F3,209 = 12.6, p < 0.001) and constructional praxis tasks, including copy of drawings, copy with landmarks, and Rey's Complex Figure-Copy were significantly lower in AD patients compared to the other groups ($Fs_{3,209}$ = 11.6–14.1, ps < 0.001).

Table S2. One-way ANOVA results were obtained on the performance of the participants on neuropsychological testing.

	**AD**	**a-MCI**	**SCD**	**HS**	**Group effect**
<u>**Verbal episodic memory**</u>					
**15-Word List:**					
Immediate recall (cut-off <u>></u>28.5)	26.6 (7.9)*#$	34.1 (8.7)£ &	46.3 (8.2)§	51.8 (7.8)	$F_{3,209}$=77.1 **p<0.001**
Delayed recall (cut-off <u>></u>4.6)	2.0 (2.9)*#$	5.6 (2.9) £&	9.8 (2.8) §	11.7 (2.3)	$F_{3,209}$=96.2 **p<0.001**
Recognition *hit rates*	9.1 (3.7)*#$	11.4 (3.4) £&	14.1 (1.0)	14.6 (1.0)	$F_{3,209}$=33.1 **p<0.001**

Recognition *false*	9.5 (6.8)* #$	4.0 (4.5) £&	0.7 (1.0)	0.5 (1.0)	$F_{3,209}$=38.5 **p<0.001**
**Short Story:**					
Immediate recall (cut-off > 3.1)	2.9 (2.2)*#$	4.3(2.0) £&	6.4 (1.1)	6.8 (0.8)	$F_{3,209}$=45.5 **p<0.001**
Delayed recall (cut-off ≥ 2.8)	1.9 (2.3)*#$	3.5 (2.2)&	6.1 (1.0)	6.6 (0.8)	$F_{3,209}$=57.9 **p<0.001**
**Visuo-spatial episodic memory**					
**Rey's Complex Figure:**					
Immediate recall (cut-off > 6.4)	6.9 (5.4)*#$	10.6 (5.2)&	17.6 (4.7)	17.0 (3.9)	$F_{3,209}$=37.5 **p<0.001**
Delayed recall (cut-off ≥ 6.3)	5.3 (5.5)*#$	10.0 (4.4)&£	17.2 (5.9)	16.4 (4.7)	$F_{3,209}$=38.6 **p<0.001**

**Verbal short-term memory**					
**Digit Span forward** (cut-off > 3.7)	5.5 (1.1)*	5.7 (0.8) &	6.0 (1.0)	6.3 (1.2)	$F_{3,209}$=4.9 **p<0.001**
**Digit Span backward**	3.5 (1.5)*	3.8 (0.9)#*	4.5 (1.0)&§	4.7 (0.8)&	$F_{3,209}$=9.4 **p<0.001**
**Visuo-spatial short-term memory**					
**Corsi Span forward** (cut-off $\geq$ 3.5)	3.4 (1.2) * #$	4.8 (0.8)$	5.5 (1.0)	5.7 (0.9)	$F_{3,209}$=16.4 **p<0.001**
**Corsi Span backward**	2.7 (1.6) * #$	4.2 (1.4)&	4.9 (0.9)	5.2 (1.0)	$F_{3,209}$=21.0 **p<0.001**

**<u>Executive functions</u>**					
**Phonological verbal fluency** (cut-off ≥ 17.3)	26.5 (8.9)*#$	32.1 (11.2)&	37.4 (8.6)	42.5 (10.1)	$F_{3,209}$=19.1 **p<0.001**
**Modified Card Sorting Test:**					
Criteria achieved (cut-off ≥4.25)	2.6 (2.0)*#$	4.1 (1.7) &£	5.9 (0.2)	5.9 (0.3)	$F_{3,209}$=40.2 **p<0.001**
**<u>Reasoning</u>**					
**Raven's Progressive Matrices** (cut-off ≥ 18.9)	24.1 (6.2)*§	26.3 (4.4) &£	31.3 (2.6)	31.6 (2.6)	$F_{3,209}$=25.2 **p<0.001**

**Language**					
**Naming of objects** (cut-off ≥ 22)	25.2 (6.9)*#$	28.5 (2.0)	29.5 (0.8)	29.7 (0.5)	$F_{3,209}$=12.6 **p<0.001**
**Constructional praxis**					
**Copy of drawings** (cut-off ≥ 7.1)	8.1 (2.2)*#$	9.5 (1.7)	10.1 (1.1)	9.8 (1.0)	$F_{3,209}$=11.6 **p<0.001**
**Copy of drawings with landmarks** (cut-off ≥ 61.8)	63.1 (6.5)*#$	66.9 (3.5)	69.1 (1.6)	68.1 (1.6)	$F_{3,209}$=14.1 **p<0.001**

**Rey's Complex Figure-Copy (**cut-off >23.7**)**	22.3 (10.3)*#$	28.2 (8.6)£	32.7 (3.2)	30.6 (3.9)	$F_{3,209}$=12.7 **p<0.001**

One-way ANOVA; Post-hoc comparisons: *AD *vs.* HS p-value<0.05; #AD *vs*. a-MCI p-value<0.05; $AD *vs.* SCD p-value<0.05; &a-MCI *vs.* HS p-value<0.05; £a-MCI *vs.* SCD p-value<0.05. § SCD *vs.* HS p-value<0.05

Abbreviations: AD: Alzheimer's Disease; a-MCI: amnestic Mild Cognitive Impairment; HS: healthy subjects . See text for further details.

Regression analyses

In Table S3 are detailed the results of the regression analyses

Table S3 Results of the Regression analyses

			Coefficients of the regression model							
Entire sample	***Dependent variable***	***Independent Variables***	***B***	***Std. Err***	***Beta***	***t***	***p-level***	***95%IC***		***VIF***
								Lower limit	*Upper limit*	
	MMSE	*Age*	*-0.047*	*0.024*	*-0.11*	*-2.02*	*0.045*	*-0.094*	*-0.001*	*1.65*
		CR	*0.126*	*0.325*	*0.034*	*0.388*	*0.699*	*-0.516*	*0.768*	*4.64*
		OTIN	*-0.0001*	*0.0001*	*0.001*	*0.009*	*0.993*	*0.000*	*0.000*	*5.96*
		PreCiN	*-0.0001*	*0.0001*	*0.008*	*0.071*	*0.943*	*0.000*	*0.000*	*8.18*
		CHiN	*-0.0001*	*0.0001*	*-0.021*	*-0.284*	*0.777*	*0.000*	*0.000*	*3.33*
		AD(dummy)	*-6.367*	*0.535*	*-0.781*	*-11.898*	***<0.001***	*-7.423*	*-5.311*	*2.53*
		a-MCI(dummy)	*-1.977*	*0.478*	*-0.242*	*-4.134*	***<0.001***	*-2.921*	*-1.033*	*2.02*
		SCD(dummy)	*0.317*	*0.507*	*0.036*	*0.625*	*0.533*	*-0.683*	*1.317*	*1.94*
		CRxAD	*0.341*	*0.436*	*0.049*	*0.783*	*0.435*	*-0.519*	*1.202*	*2.28*
		CRxa-MCI	*0.464*	*0.435*	*0.067*	*1.066*	*0.288*	*-0.395*	*1.323*	*2.31*
		CRxSCD	*-0.159*	*0.465*	*-0.020*	*-0.343*	*0.732*	*-1. 000*	*0.758*	*2.01*
		OTINxAD	*-0.0001*	*0.0001*	*-0.078*	*-0.977*	*0.330*	*0.000*	*0.000*	*3.75*
		OTINxa-MCI	*-0.0001*	*0.0001*	*-0.007*	*-0.103*	*0.918*	*0.000*	*0.000*	*2.57*
		OTINxSCD	*-0.0001*	*0.0001*	*-0.025*	*-0.354*	*0.724*	*0.000*	*0.000*	*2.97*
		PreCiNxAD	*0.0001*	*0.0001*	*0.125*	*1.545*	*0.124*	*0.000*	*0.000*	*3.87*
		PreCiNxa-MCI	*0.0001*	*0.0001*	*0.040*	*0.507*	*0.613*	*0.000*	*0.000*	*3.74*

		PreCiNxSCD	*-0.0001*	*0.0001*	*-0.004*	*-0.053*	*0.958*	*0.000*	*0.000*	*4.06*
		CHiNxAD	*-0.0001*	*0.0001*	*-0.021*	*-0.376*	*0.707*	*0.000*	*0.000*	*1.83*
		CHiNxa-MCI	*-0.0001*	*0.0001*	*-0.059*	*-1.063*	*0.289*	*0.000*	*0.000*	*1.78*
		CHiNxSCD	*-0.0001*	*0.0001*	*-0.009*	*-0.156*	*0.876*	*0.000*	*0.000*	*1.87*
	MeCS	*Age*	*-0.029*	*0.006*	*-0.243*	*-4.604*	*0.141*	*-0.046*	*0.317*	*1.73*
		CR	*0.136*	*0.092*	*0.132*	*1.480*	*0.372*	*-0.127*	*0.048*	*4.99*
		OTIN	*-0.0001*	*0.0001*	*0.046*	*0.468*	*0.641*	*0.000*	*0.000*	*6.12*
		PreCiN	*-0.0001*	*0.0001*	*-0.012*	*-0.107*	*0.915*	*0.000*	*0.000*	*7.41*
		CHiN	*-0.0001*	*0.0001*	*-0.027*	*-0.301*	*0.764*	*0.000*	*0.000*	*4.95*
		AD(dummy)	*-1.860*	*0.150*	*-0.794*	*-12.440*	***<0.001***	*-2.155*	*-1.564*	*2.54*
		a-MCI(dummy)	*-1.081*	*0.123*	*-0.494*	*-8.794*	***<0.0010***	*-1.324*	*-0.838*	*1.97*
		SCD(dummy)	*-0.203*	*0.145*	*-0.086*	*-1.398*	*0.164*	*-0.491*	*0.084*	*2.36*
		CRxAD	*-0.258*	*0.125*	*-0.126*	*-2.070*	***0.040***	*-5.505*	*-0.012*	*2.30*
		CRxa-MCI	*-0.276*	*0.121*	*-0.143*	*-2.289*	***0.024***	*-0.515*	*-0.038*	*2.45*
		CRxSCD	*-0.157*	*0.127*	*-0.074*	*-1.233*	*0.220*	*-0.408*	*0.095*	*2.25*
		OTINxAD	*0.0001*	*0.0001*	*0.105*	*1.295*	*0.197*	*0.000*	*0.000*	*4.08*
		OTINxa-MCI	*-0.0001*	*0.0001*	*-0.020*	*-0.293*	*0.770*	*0.000*	*0.000*	*3.04*
		OTINxSCD	*-0.0001*	*0.0001*	*-0.015*	*-0.232*	*0.817*	*0.000*	*0.000*	*2.47*
		PreCiNxAD	*-0.0001*	*0.0001*	*-0.090*	*-1.162*	*0.247*	*0.000*	*0.000*	*3.78*
		PreCiNxa-MCI	*-0.0001*	*0.0001*	*-0.022*	*-0.286*	*0.775*	*0.000*	*0.000*	*3.77*
		PreCiNxSCD	*0.0001*	*0.0001*	*0.027*	*0.354*	*0.724*	*0.000*	*0.000*	*3.54*
		CHiNxAD	*0.0001*	*0.0001*	*0.050*	*0.869*	*0.386*	*0.000*	*0.000*	*2.04*
		CHiNxa-MCI	*0.0001*	*0.0001*	*0.042*	*0.655*	*0.514*	*0.000*	*0.000*	*2.58*
		CHiNxSCD	*-0.0001*	*0.0001*	*-0.005*	*-0.075*	*0.940*	*0.000*	*0.000*	*3.34*

Internetworks correlations

In Table S4 are shown the Pearson's coefficients and the p-values of the inter-networks correlations observed in the participants

Table S4 inter-networks correlations

**Groups**		**OTIN**	**PreCiN**	**CHiN**
**Entire Sample**				
	**OTIN**	-	r=0.57, p<0.00001	r=0.11, p=0.112
	**PreCiN**	r=0.57, p<0.00001	-	r=0.135, p=0.051
	**CHiN**	r=0.11, p=0.112	r=0.135, p=0.051	-
**AD**				
	**OTIN**	-	r=0.410, p=0.002	r=0.212, p=0.113
	**PreCiN**	r=0.410, p=0.002	-	r=0.08, p=0.56
	**CHiN**	r=0.212, p=0.113	r=0.08, p=0.56	-
**a-MCI**				
	**OTIN**	-	r=0.52, p<0.00001	r=0.03, p=0.793
	**PreCiN**	r=0.52, p<0.00001	-	r=0.08, p=0.537
	**CHiN**	r=0.03, p=0.793	r=0.08, p=0.537	-
**SCD**				
	**OTIN**	-	r=0.67, p<0.00001	r=0.11, p=0.469
	**PreCiN**	r=0.67, p<0.00001	-	r=0.23, p=0.127
	**CHiN**	r=0.11, p=0.469	r=0.23, p=0.127	-
**HS**				
	**OTIN**	-	r=0.43, p=0.002	r=0.17, p=0.906
	**PreCiN**	r=0.43, p=0.002	-	r=0.07, p=0.619
	**CHiN**	r=0.17, p=0.906	r=0.07, p=0.619	

Correlations between structural networks, CR level, or cognitive performances.

Table S5

Groups		OTIN	PreCiN	CHiN
**Entire Sample**				
	**CR level**	r=0.06, p=0.368	r=-0.02, p=0.813	r=0.08, p=0.226
	**MMSE**	r=0.31, p<0.00001	r=0.42, p=<0.00001	r=0.02, p=0.733
	**MeCS**	r=0.40, p<0.00001	r=0.42, p=<0.00001	r=0.22, p=0.006
**AD**				
	**CR level**	r=0.11, p=0.407	r=-0.06, p=0.629	r=0.167, p=0.215
	**MMSE**	r=-0.03, p=0.818	r=0.16, p=0.248	r=-0.05, p=0.735
	**MeCS**	r=0.356, p=0.028	r=-0.06, p=0.698	r=0.24, p=0.147
**a-MCI**				
	**CR level**	r=0.01, p=0.958	r=-0.14, p=0.293	r=-0.03, p=0.780
	**MMSE**	r=0.07, p=0.599	r=0.15, p=0.272	r=-0.173, p=0.214
	**MeCS**	r=0.04, p=0.788	r=0.08, p=0.581	r=0.24, p=0.105
**SCD**				
	**CR level**	r=0.09, p=0.556	r=0.13, p=0.390	r=-0.10 p=0.526
	**MMSE**	r=-0.20, p=0.193	r=-0.10, p=0.524	r=0.01, p=0.949
	**MeCS**	r=0.17 p=0.300	r=0.26 p=0.120	r=0.22, p=0.192
**HS**				
	**CR level**	r=0.06 p=0.696	r=0.07 p=0.611	r=0.29 p=0.045
	**MMSE**	r=0.13 p=0.372	r=0.19 p=0.190	r=0.01 p=0.987
	**MeCS**	r=0.28 p=0.085	r=0.27 p=0.107	r=0.24 p=0.139

Moderation analyses.

General cognitive efficiency

In the AD group no significant global effects were found between MMSE scores, GMC of OTIN, PreCiN and CHiN, and CR level: i) model including X=OTIN, Y=MMSE and M=CR level: R=0.26, $F_{5,203}$=0.67, p=0.646; ii) model including X=PreCiN, Y=MMSE and M=CR Level: R=0.34, $F_{5,203}$=1.23, p=0.307; iii) model including X=CHiN, Y=MMSE and M=CR Level: R=0.30, $F_{5,203}$=0.91, p=0.479; in the a-MCI group no significant global effects were found between MMSE scores, GMC of OTIN, PreCiN and CHiN, and CR level: i) model including X=OTIN, Y=MMSE and M=CR level: R=0.50 $F_{5,203}$=3.23, p=0.137; ii) model including X=PreCiN, Y=MMSE and M=CR Level: R=0.41, $F_{5,203}$=1.94, p=0.105; iii) model including X=CHiN, Y=MMSE and M=CR Level: R=0.47, $F_{5,203}$=2.66, p=0.337. Table S6 reports the effects of the models of the moderation analyses obtained by groups.

Table S6. Moderation analyses for general cognitive efficiency (MMSE score)

	Effects of the model			
	Predictors	***Beta coefficient***	***t***	***p-values***
GROUP				

AD				
	OTIN	*0.01*	*-0.23*	*0.817*
	CR Level	*0.32*	*0.67*	*0.503*
	OTIN x CR Level	*0.01*	*0.44*	*0.661*
	PreCiN	*0.01*	*1.34*	*0.186*
	CR Level	*0.28*	*0.61*	*0.542*
	PreCiN x CR Level	*0.01*	*-1.00*	*0.318*
	CHiN	*0.01*	*-0.23*	*0.821*
	CR Level	*0.36*	*0.75*	*0.451*
	CHiN x CR Level	*0.01*	*-1.02*	*0.312*
a-MCI				
	OTIN	*0.01*	*-0.81*	*0.419*
	CR Level	*0.28*	*0.97*	*0.335*
	OTIN x CR Level	*0.01*	*2.53*	*0.015*
	PreCiN	*0.01*	*0.36*	*0.721*
	CR Level	*0.54*	*1.83*	*0.073*
	PreCiN x CR Level	*0.01*	*0.85*	*0.399*

	CHiN	*0.01*	*-1.90*	*0.063*
	CR Level	*0.47*	*1.69*	*0.096*
	CHiN x CR Level	*0.01*	*0.02*	*0.985*
SCD				
	OTIN	*0.01*	*-1.66*	*0.105*
	CR Level	*0.02*	*0.20*	*0.843*
	OTIN x CR Level	*0.01*	*0.949*	*0.349*
	PreCiN	*0.01*	*-0.24*	*0.814*
	CR Level	*-0.02*	*-0.10*	*0.917*
	PreCiN x CR Level	*0.01*	*-1.13*	*0.266*
	CHiN	*0.01*	*-0.36*	*0.722*
	CR Level	*0.01*	*0.09*	*0.923*
	CHiN x CR Level	*0.01*	*-1.19*	*0.240*
HS				
	OTIN	*0.01*	*0.71*	*0.483*
	CR Level	*0.18*	*1.20*	*0.238*
	OTIN x CR Level	*0.01*	*0.13*	*0.897*

PreCiN	*0.01*	*1.05*	*0.298*
CR Level	*0.17*	*1.16*	*0.251*
PreCiN x CR Level	*0.01*	*0.09*	*0.930*
CHiN	*0.01*	*-0.22*	*0.829*
CR Level	*0.15*	*0.99*	*0.323*
CHiN x CR Level	*0.01*	*0.65*	*0.517*

Memory performances

In the AD group, no significant global effects were found between MeCS, GMC of OTIN, PreCiN and CHiN, and CR level: i) model including X=OTIN, Y= MeCS and M=CR level: R=0.37, $F_{5,51}$=1.04, p=0.410; ii) model including X=PreCiN, Y= MeCS and M=CR Level: R=0.26, $F_{5,51}$=0.46, p=0.804; iii) model including X=CHiN, Y= MeCS and M=CR Level: R=0.35, $F_{5,51}$=0.89, p=0.495. In the a-MCI group no significant global effects were found between MeCS, GMC of PreCiN and CHiN, i) model including X=PreCiN, Y= MeCS and M=CR Level: R=0.55, $F_{5,53}$=3.51, p=0.010; ii) model including X=CHiN, Y= MeCS and M=CR Level: R=0.52, $F_{5,53}$=3.01, p=0.208. In the SCD group no significant global effects were found between MeCS, GMC of PreCiN and CHiN, i) model including X=PreCiN, Y= MeCS and M=CR Level: R=0.64, $F_{5,38}$=4.30, p=0.004 ii) model including X=CHiN, Y= MeCS and M=CR Level: R=0.63, $F_{5,38}$=4.07, p=0.058. Table S7 reports the effects of the models of the moderation analyses obtained by groups.

Table S7. Moderation analyses for Memory performance (MeCS)

	Effects of the model			
	Predictors	***Beta coefficient***	***t***	***p-values***
GROUP				
AD				

	OTIN	*0.01*	*1.82*	*0.080*
	CR Level	*-0.02*	*-0.29*	*0.770*
	OTIN x CR Level	*0.01*	*-0.33*	*0.746*
	PreCiN	*0.01*	*1.61*	*0.116*
	CR Level	*-0.02*	*-0.23*	*0.820*
	PreCiN x CR Level	*0.01*	*-0.51*	*0.612*
	CHiN	*0.01*	*-0.72*	*0.475*
	CR Level	*-0.02*	*-0.19*	*0.849*
	CHiN x CR Level	*0.01*	*-0.31*	*0.760*
a-MCI				
	PreCiN	*0.01*	*0.03*	*0.972*
	CR Level	*-0.10*	*-1.06*	*0.295*
	PreCiN x CR Level	*0.01*	*1.28*	*0.206*
	CHiN	*0.01*	*0.26*	*0.797*
	CR Level	*-0.11*	*-1.11*	*0.272*
	CHiN x CR Level	*0.01*	*0.01*	*0.994*
SCD				

	PreCiN	*0.01*	*0.27*	*0.790*
	CR Level	*-0.04*	*-0.67*	*0.507*
	PreCiN x CR Level	*0.01*	*-1.13*	*0.268*
	CHiN	*0.01*	*-0.75*	*0.458*
	CR Level	*-0.03*	*0.49*	*0.625*
	CHiN x CR Level	*0.01*	*0.15*	*0.883*
HS				
	OTIN	*0.01*	*0.71*	*0.483*
	CR Level	*0.18*	*1.20*	*0.238*
	OTIN x CR Level	*0.01*	*0.13*	*0.897*
	PreCiN	*0.01*	*1.05*	*0.298*
	CR Level	*0.17*	*1.16*	*0.251*
	PreCiN x CR Level	*0.01*	*0.09*	*0.930*
	CHiN	*0.01*	*-0.22*	*0.829*
	CR Level	*0.15*	*0.99*	*0.323*
	CHiN x CR Level	*0.01*	*0.65*	*0.517*

Figures S1-S20. Detailed visualizations of the figures are presented below. These Figures illustrate the 20 spatial components extracted from the structural MRI data using Independent Component Analysis (ICA) within the GIFT toolbox. The visualization highlights the inherent capability of SBM to decompose grey matter volume into distinct sources of covariance.

As shown, certain components (e.g., Components 1, 3, 5, 6, 8, 12, 14, 16, 17, 19) represent anatomically coherent structural networks, characterized by clusters of voxels localized within functionally related brain regions. In contrast, the remaining components exhibit fragmented or peripheral patterns, primarily representing stochastic noise or motion-related artifacts. The emergence of either neurobiologically relevant signals and artefactual components is an expected outcome and a standard feature of ICA-based procedures. In SBM pipelines, this separation is a crucial step, allowing for the isolation of robust structural networks for subsequent statistical analysis while effectively filtering out non-biological variance from the dataset.

Fig S1. Characteristic profile of Component 001, serving as a visual reference for the architecture of the 20 independent structural networks extracted. Top panel (here and in the subsequent figures): the line graph illustrates the modulation of participant-specific loading coefficients across individual subjects (x-axis), standardized as z-scores (y-axis). These values quantify the degree to which each participant expresses the spatial grey matter (GM) pattern associated with this component. Higher loading values denote a stronger expression of the structural network, reflecting relatively preserved grey matter concentration (GMC) and structural integrity. Conversely, lower or negative loading values indicate reduced GMC within the network, consistent with greater structural degeneration.
Bottom panel (here and in the subsequent figures): axial slices display the voxel-wise spatial distribution generated from the ICA source matrix. The color map (ranging from 0.0 to 9.5) represents the voxel weights, identifying distinct brain regions that exhibit coherent patterns of structural covariance in GMC.

Figure S1

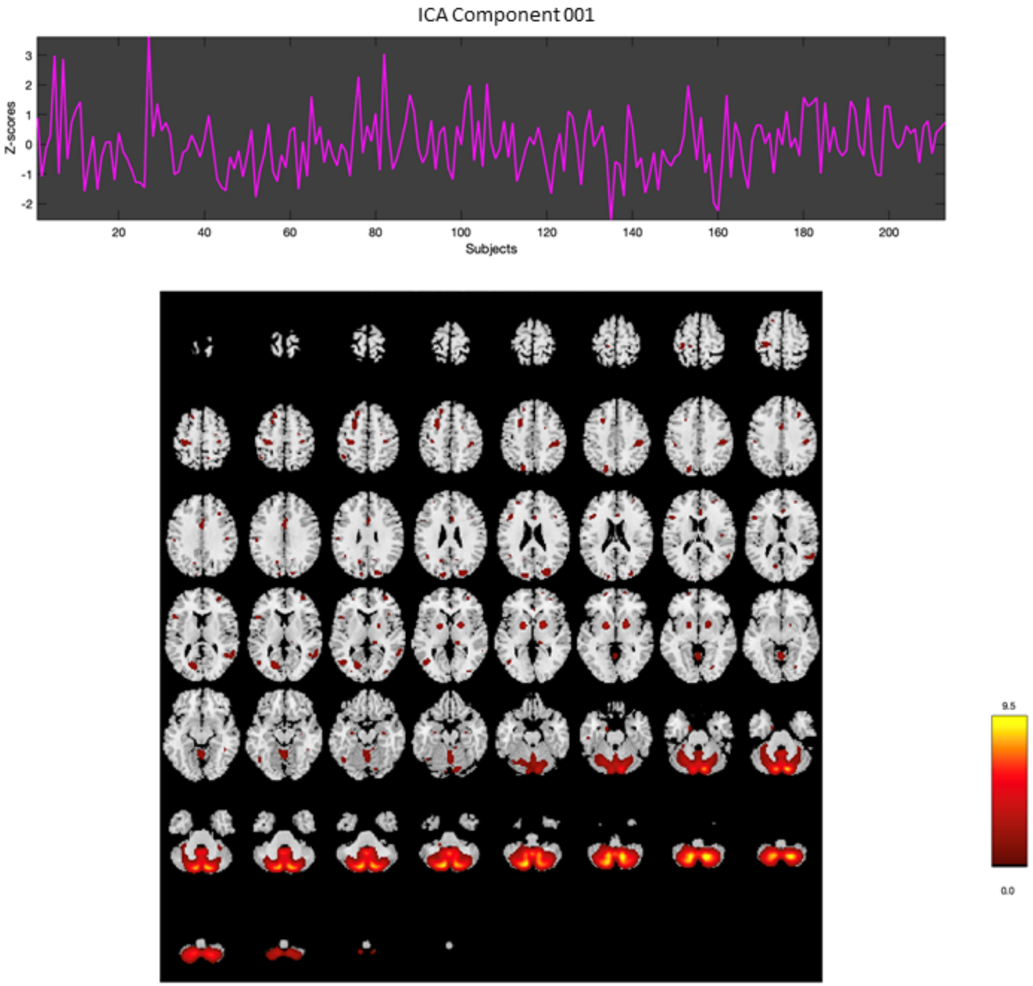

ICA Component 001
Z-scores
3
2
1
0
-1
-2
20
40
60
80
100
120
140
160
180
200
Subjects
9.5
0.0

Fig S2. Characteristic profile of Component 002

Figure S2

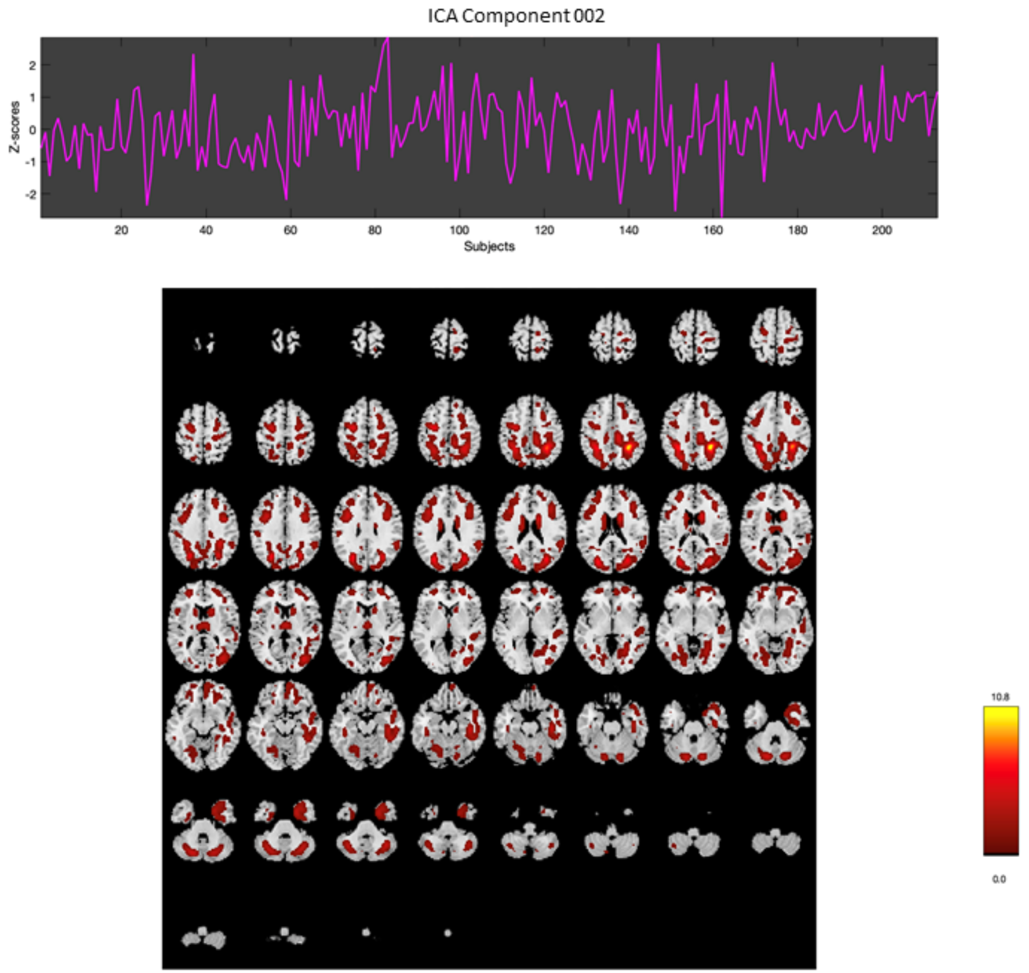

Fig S3. Characteristic profile of Component 003

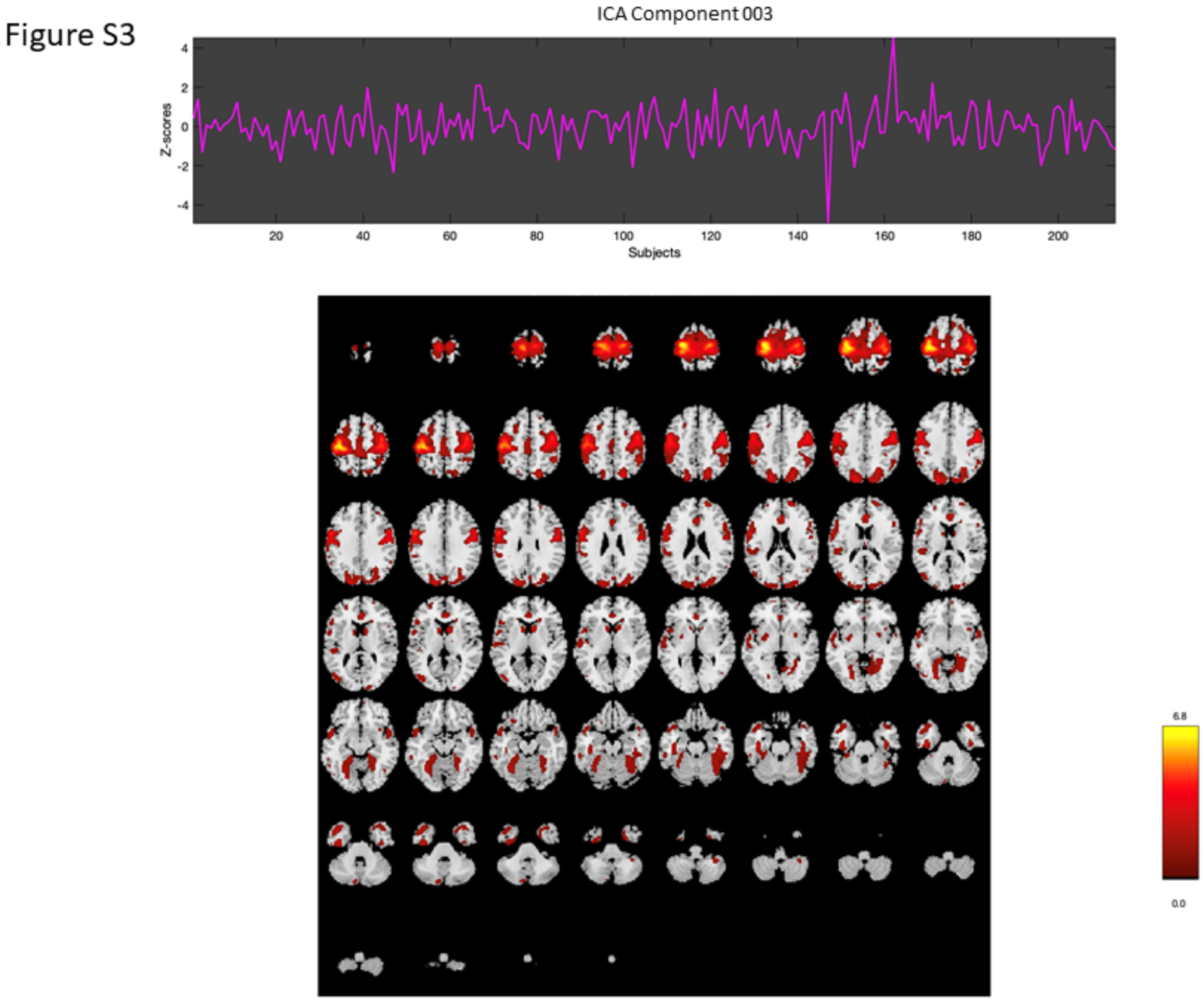

Fig S4. Characteristic profile of Component 004

Figure S4

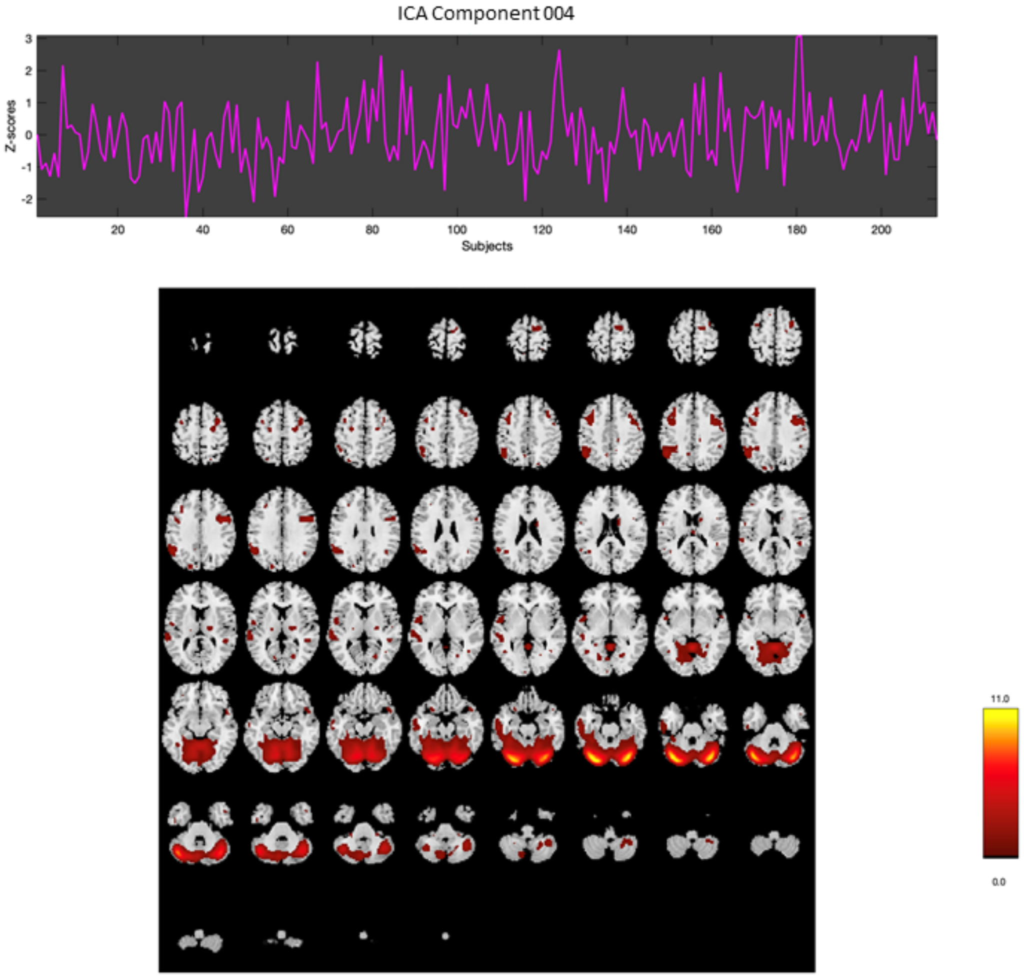

Fig S5. Characteristic profile of Component 005

Figure S5

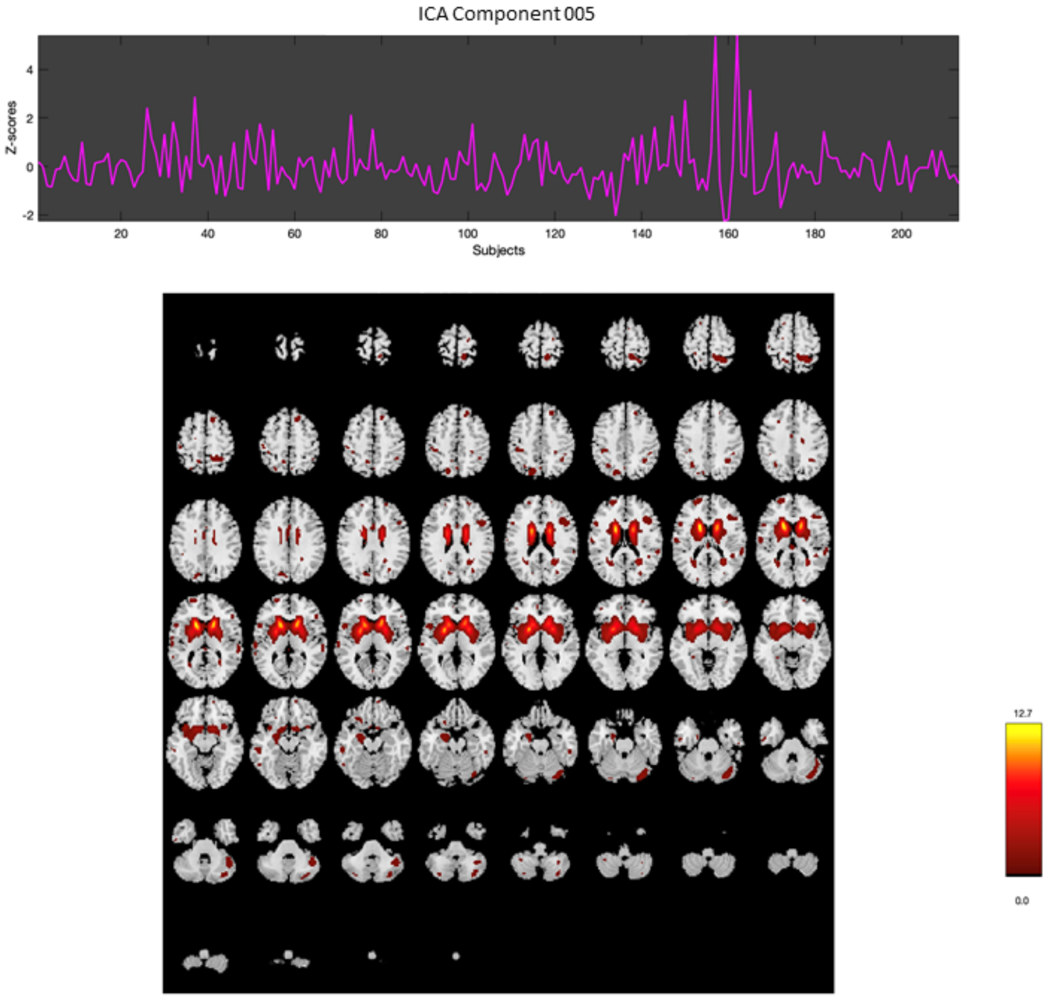

Fig S6. Characteristic profile of Component 006

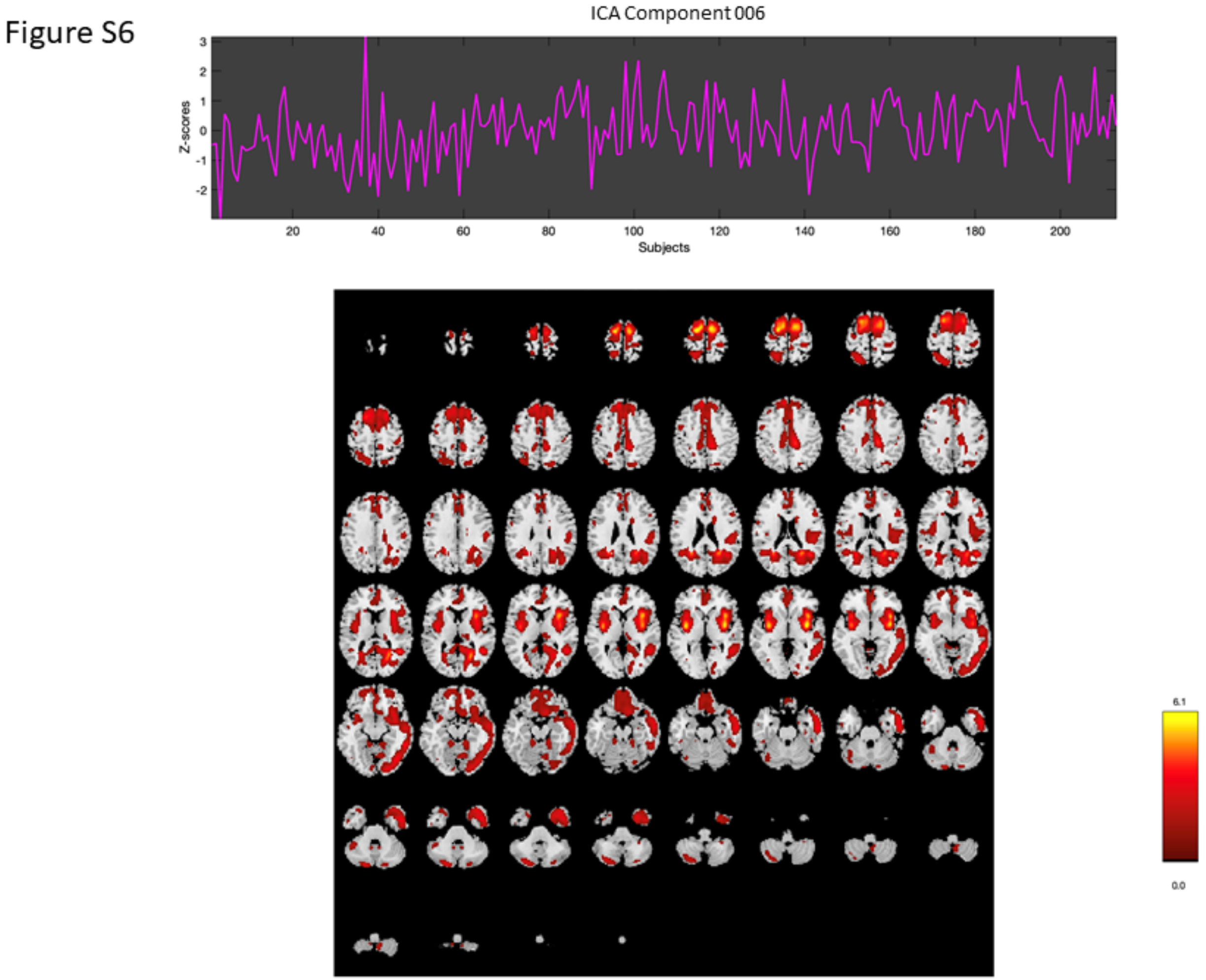

Fig S7. Characteristic profile of Component 007

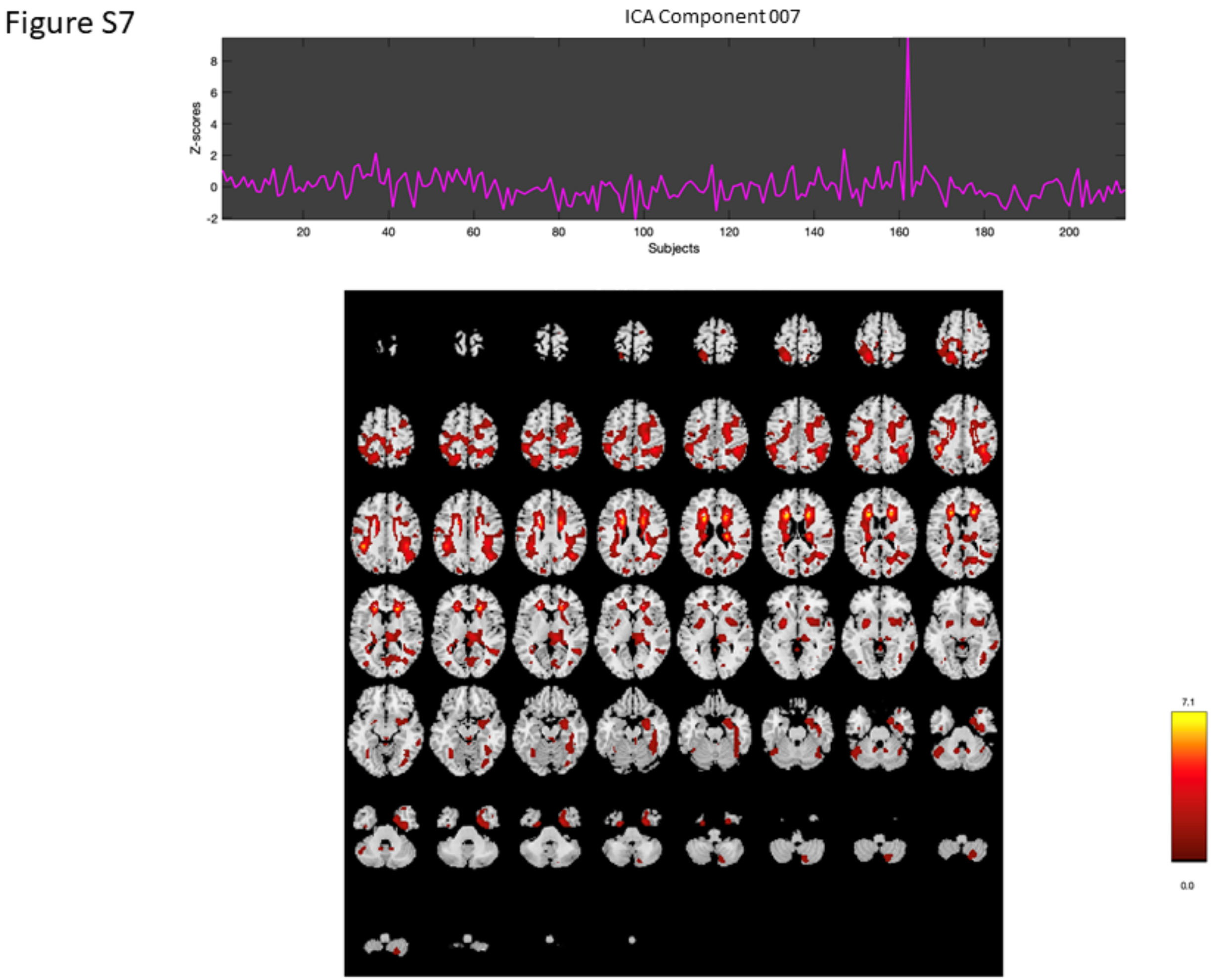

Fig S8. Characteristic profile of Component 008

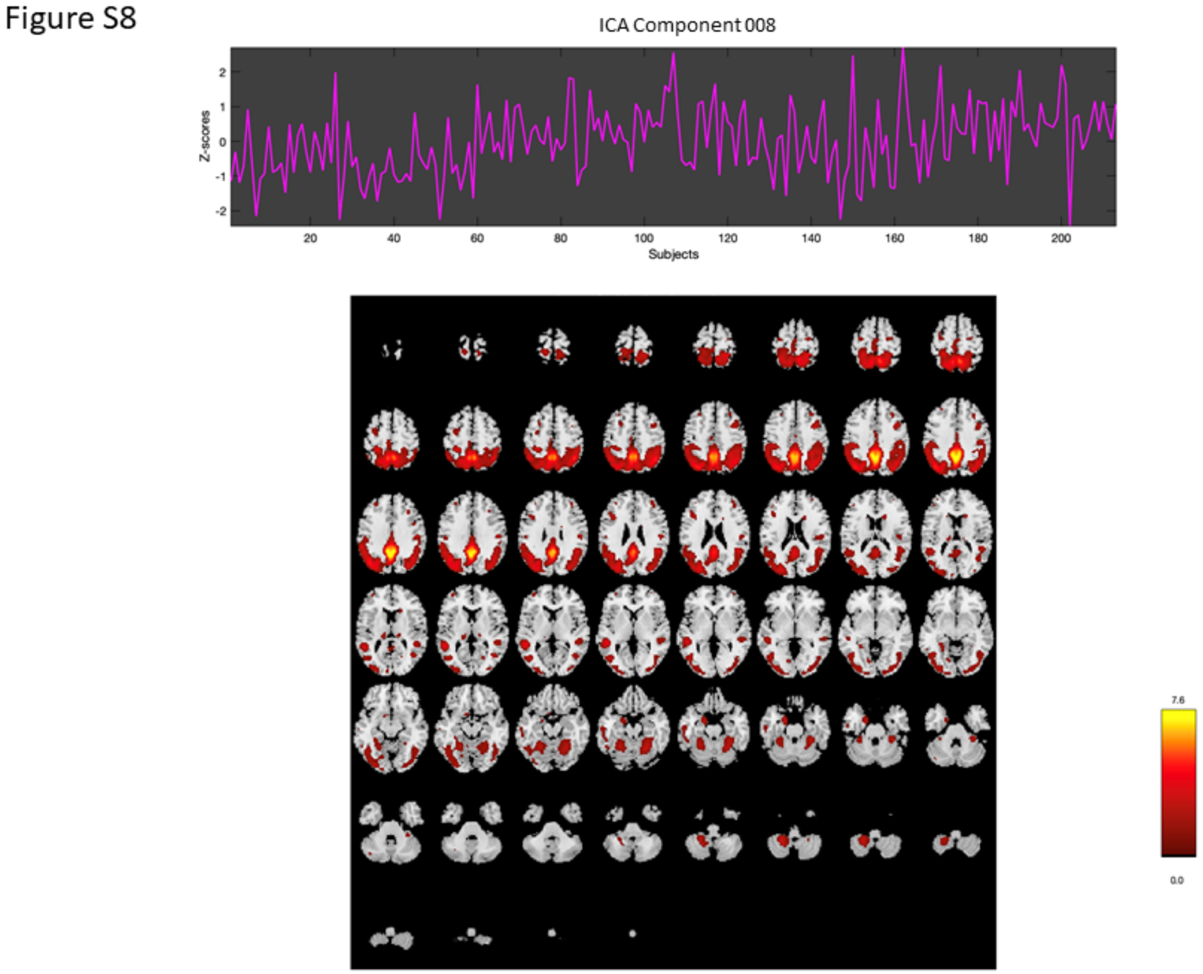

Fig S9. Characteristic profile of Component 009

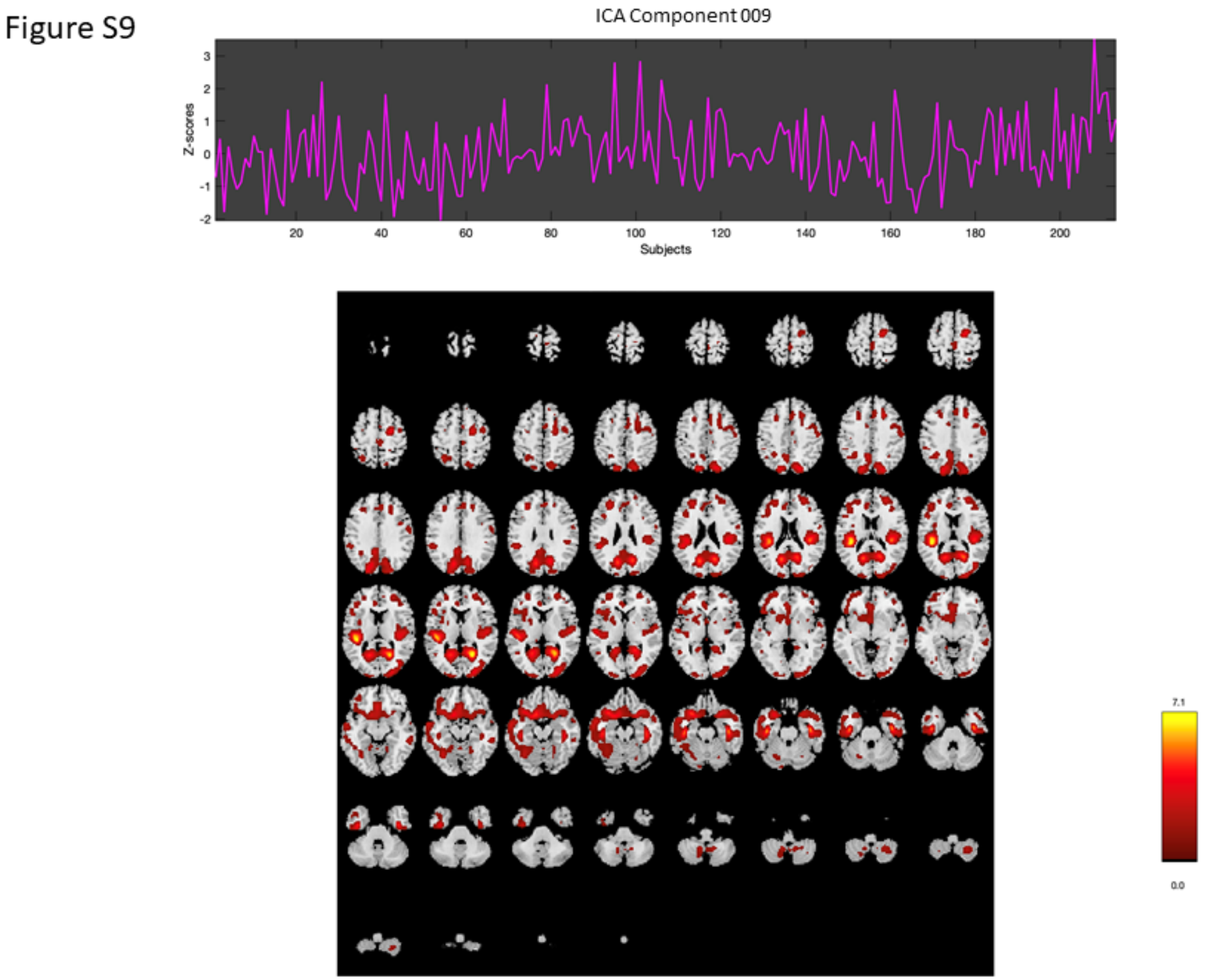

Fig S10. Characteristic profile of Component 010

Figure S10

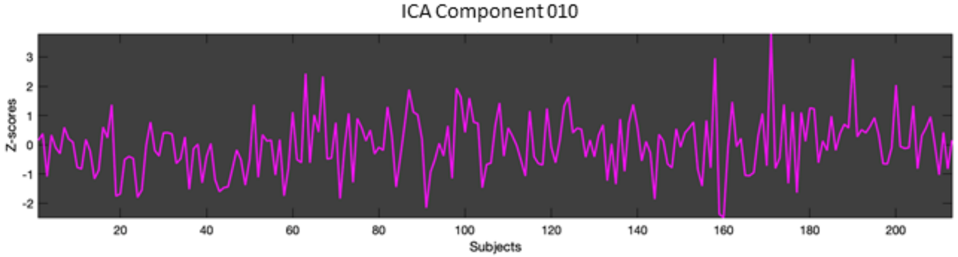

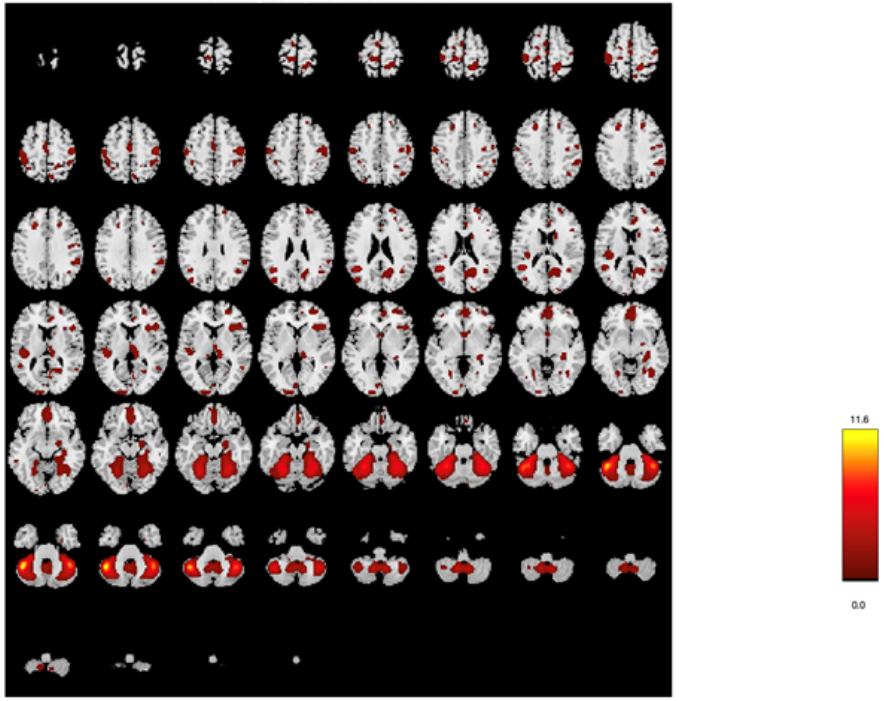

Fig S11. Characteristic profile of Component 011

Figure S11

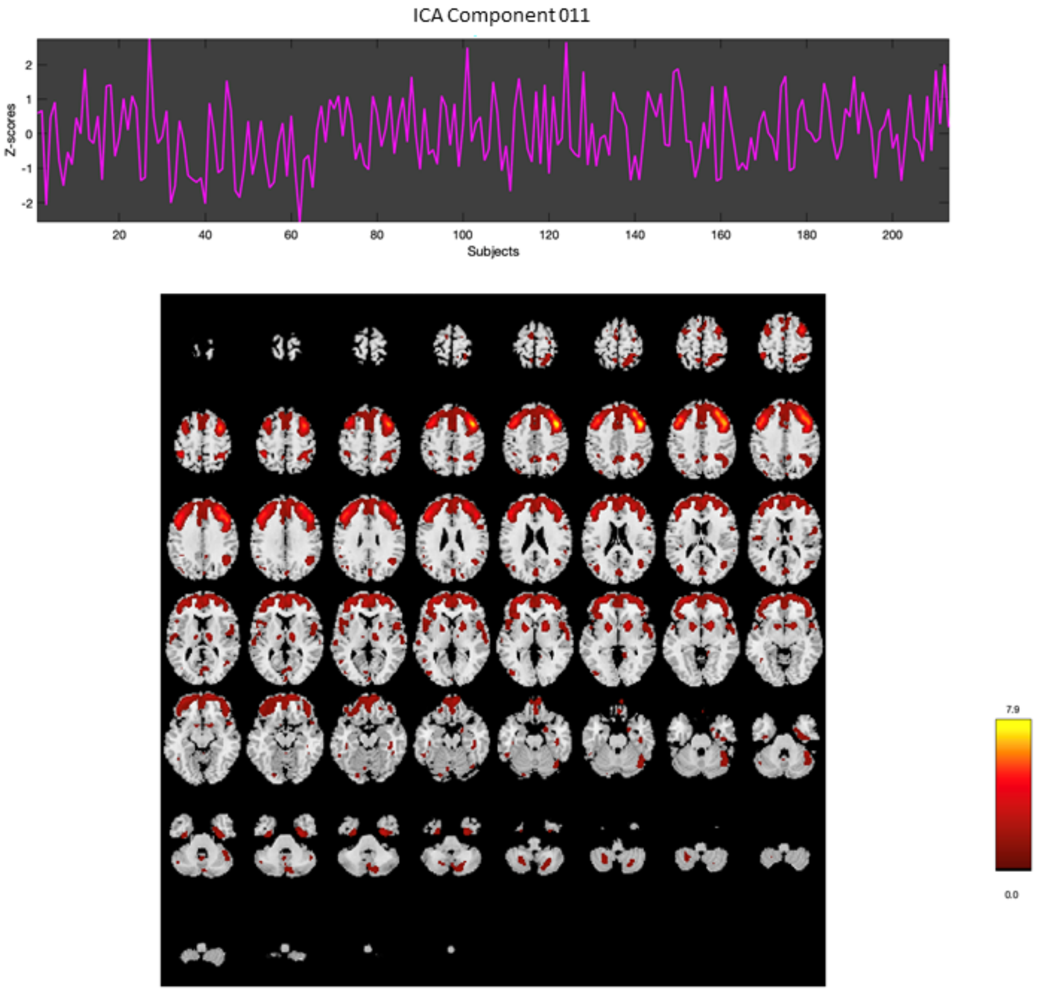

Fig S12. Characteristic profile of Component 012

Figure S12

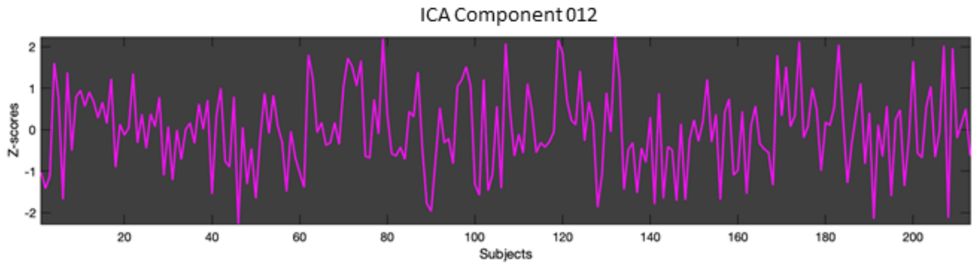

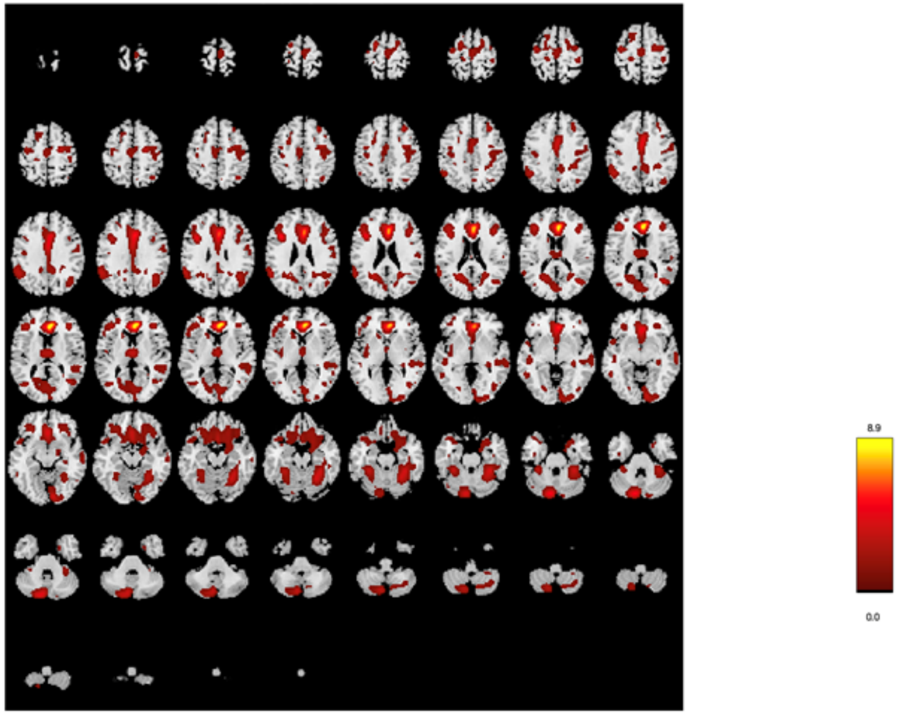

Fig S13. Characteristic profile of Component 013

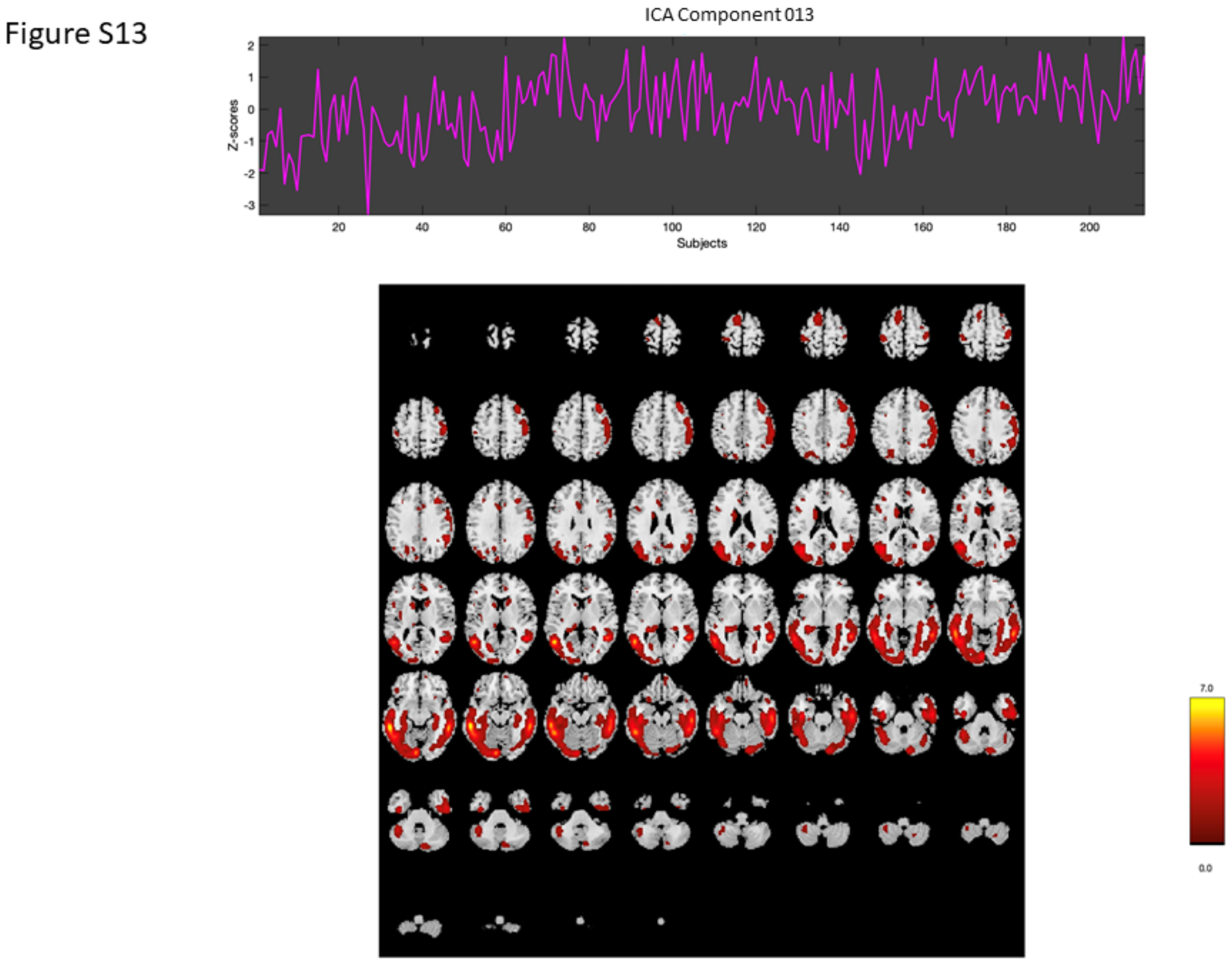

Fig S14. Characteristic profile of Component 014

Figure S14

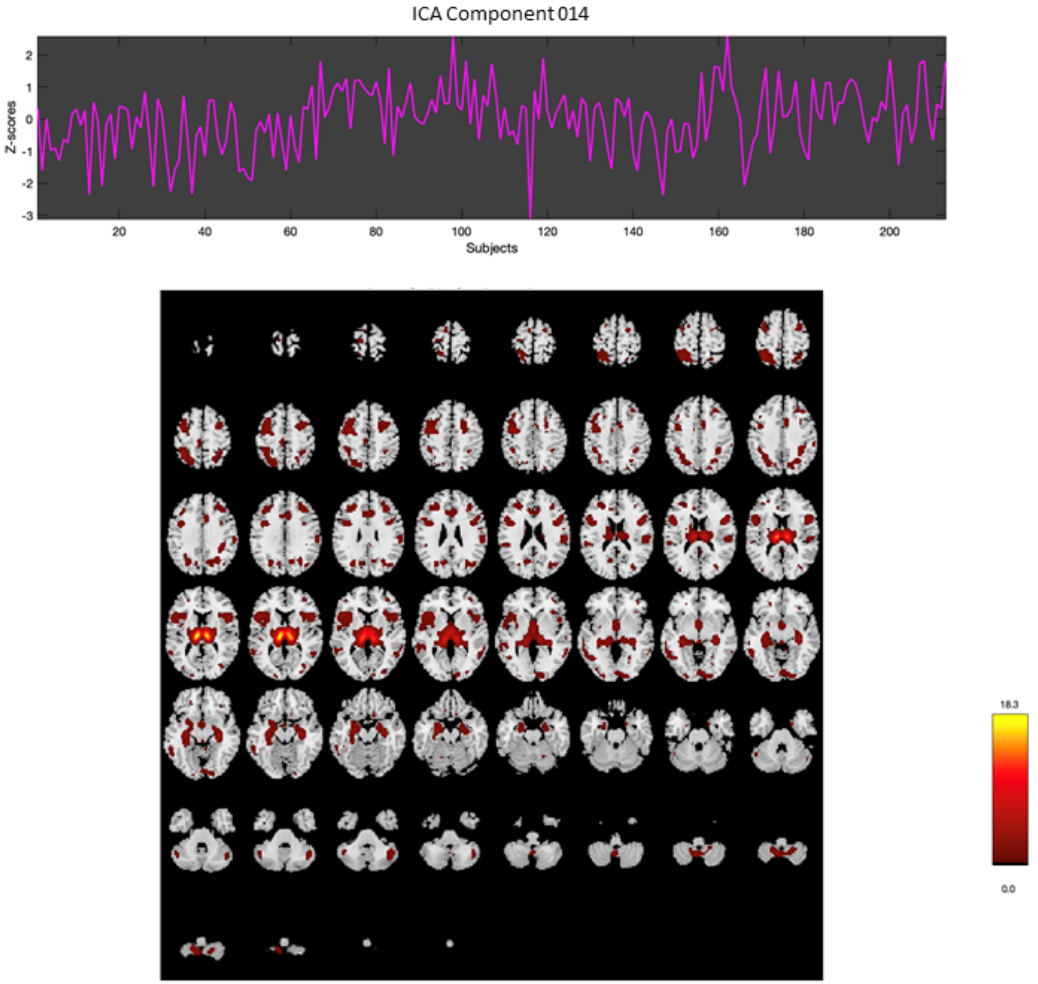

Fig S15. Characteristic profile of Component 015

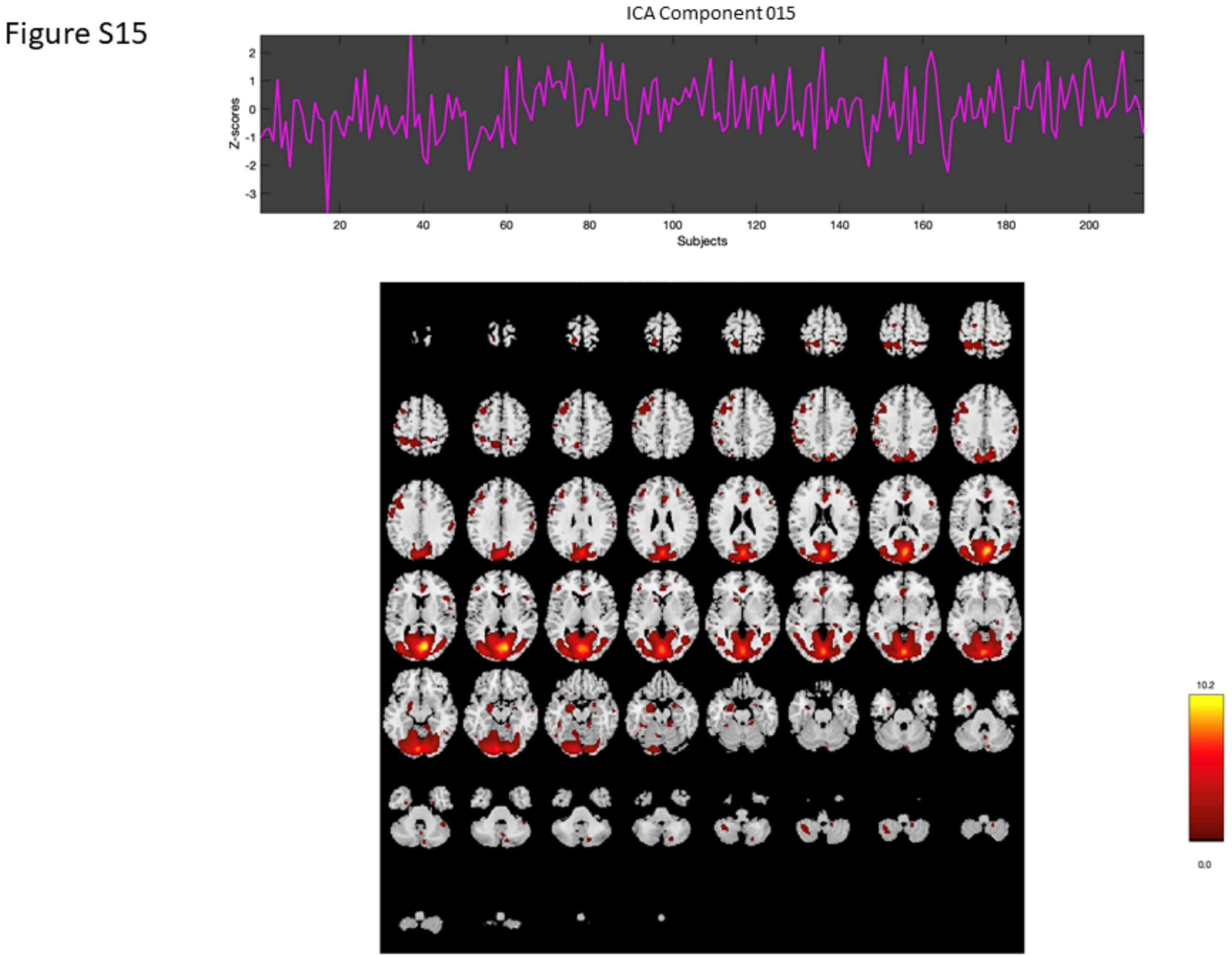

Fig S16. Characteristic profile of Component 016

Figure S16

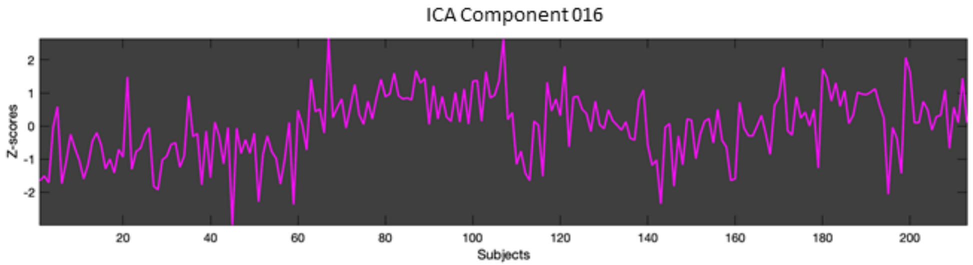

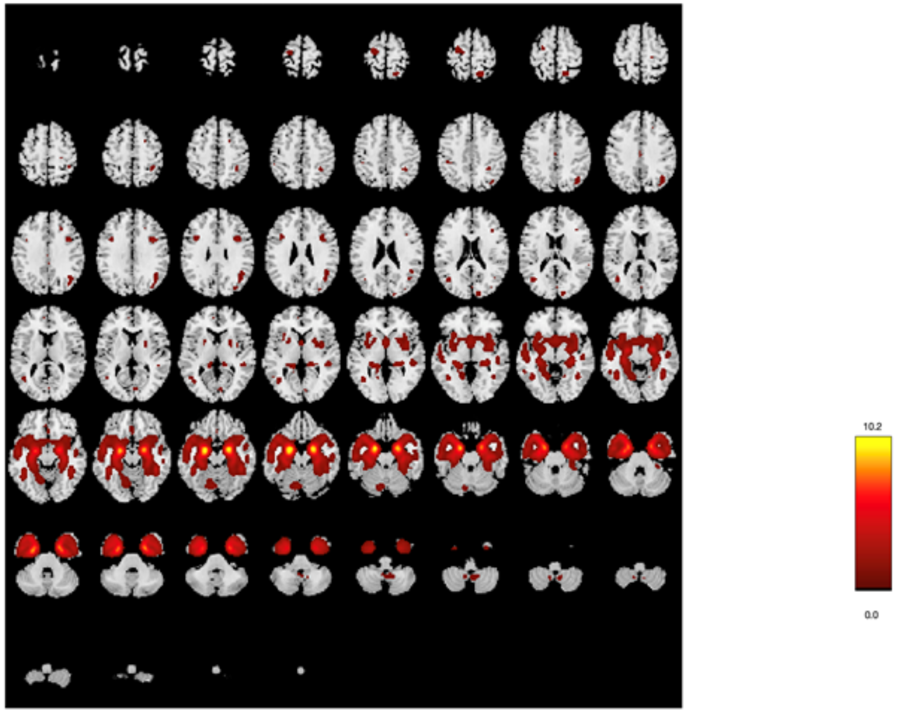

Fig S17. Characteristic profile of Component 017

Figure S17

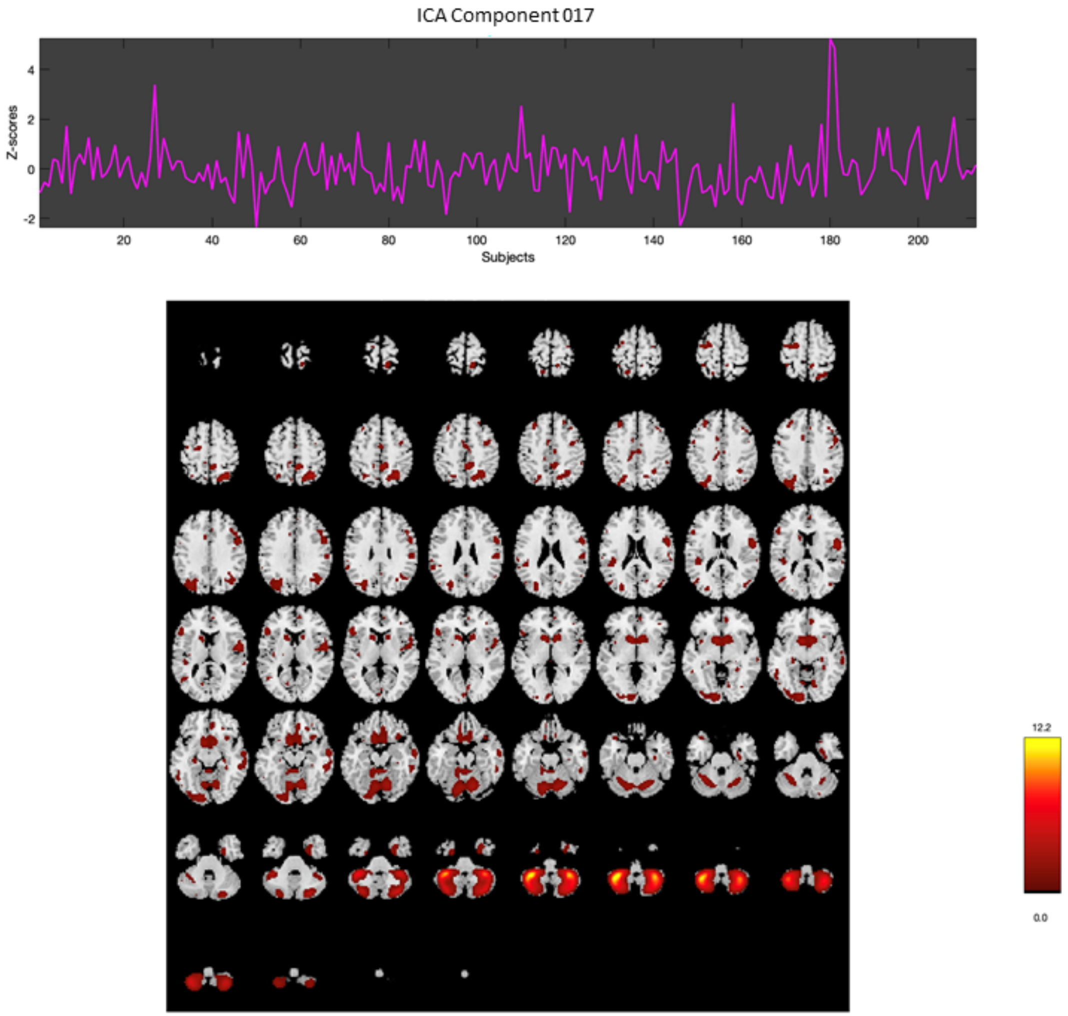

Fig S18. Characteristic profile of Component 018

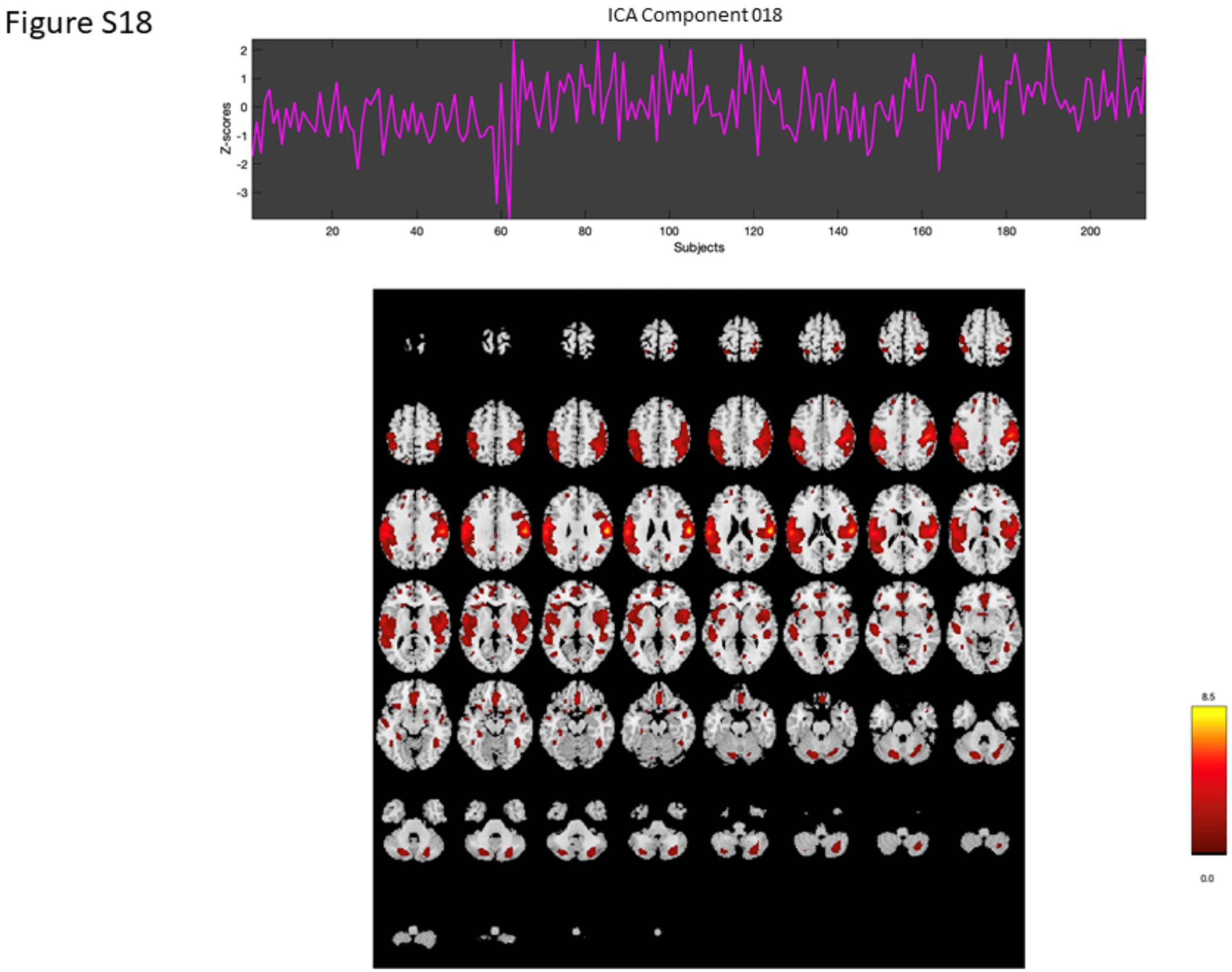

Fig S19. Characteristic profile of Component 019

Figure S19

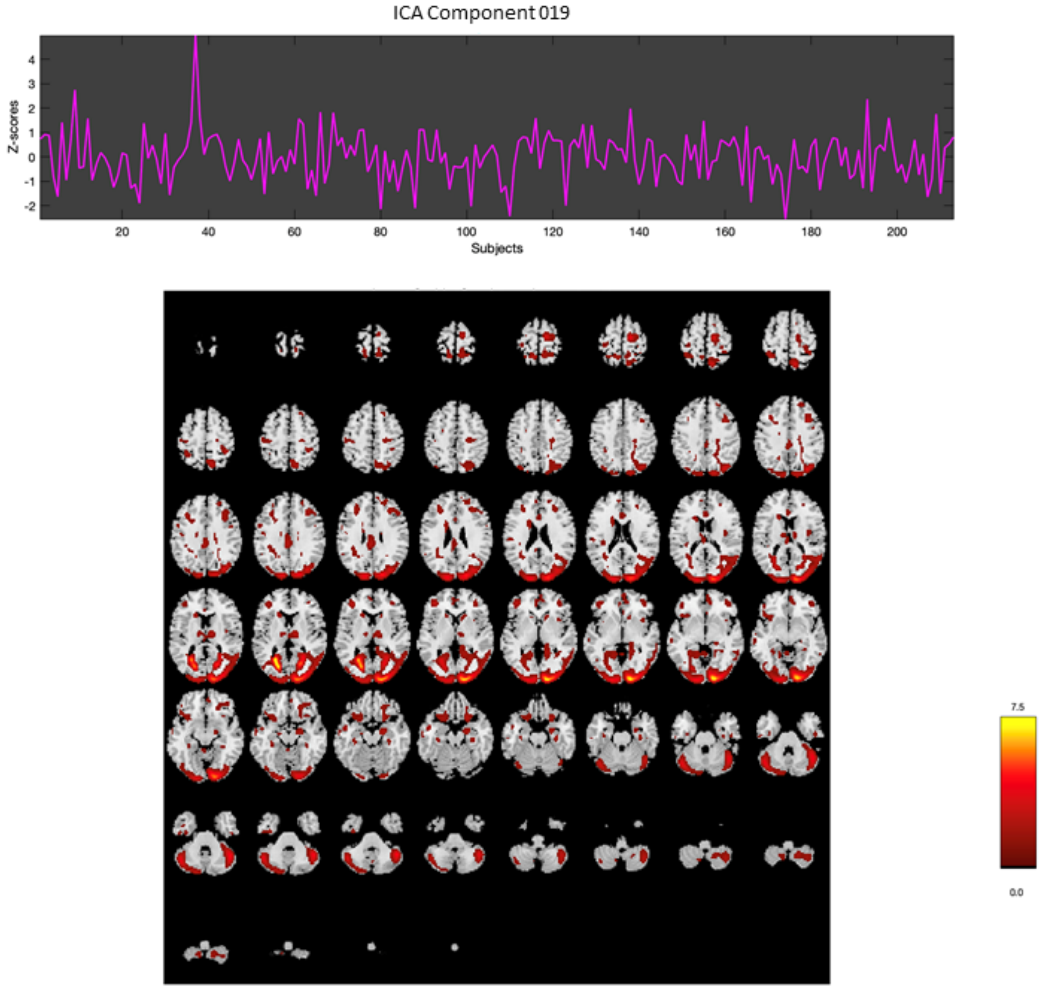

Fig S20. Characteristic profile of Component 020

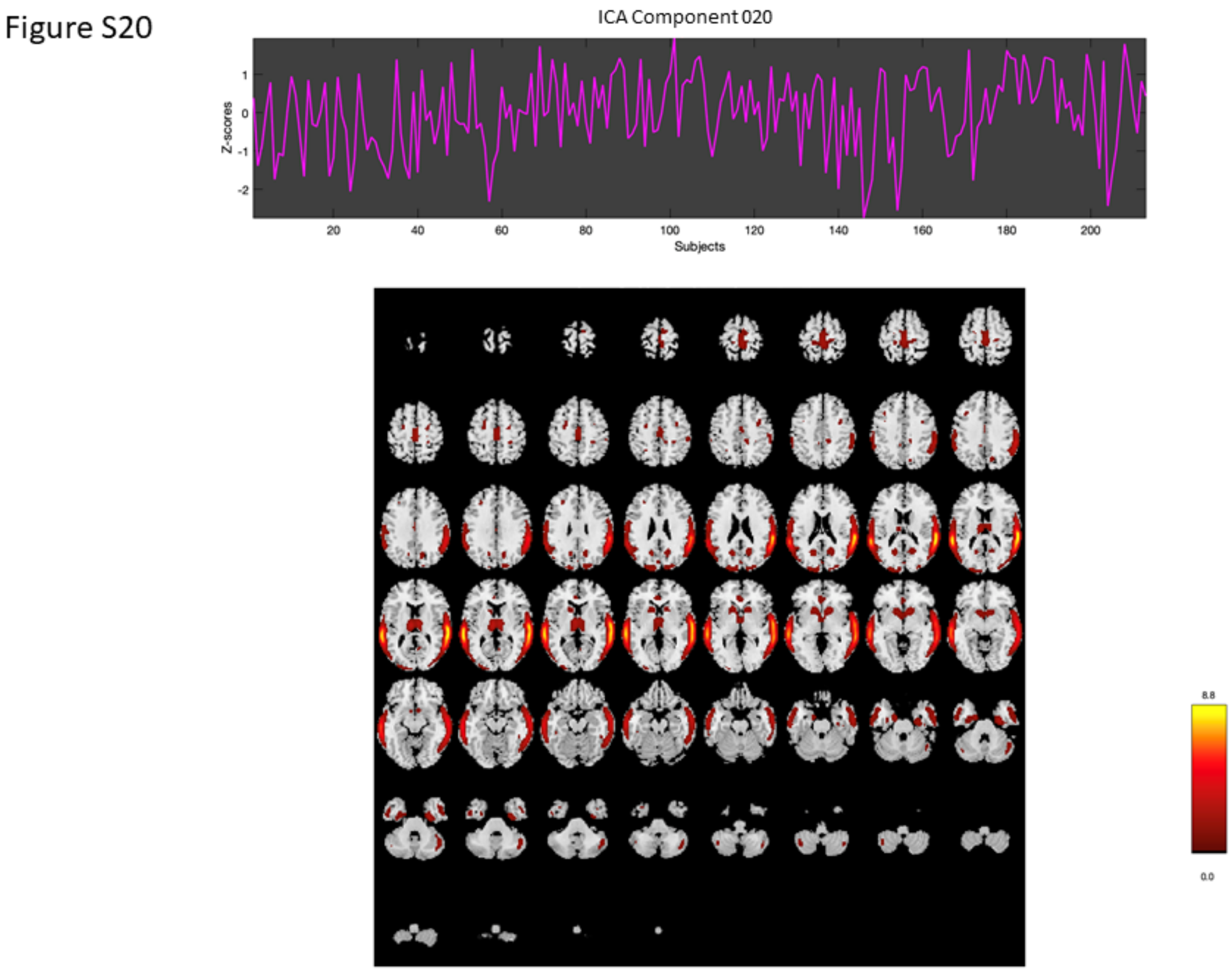

Fig. S21 Distribution of Mini-Mental State Examination (MMSE) scores across the four diagnostic groups.

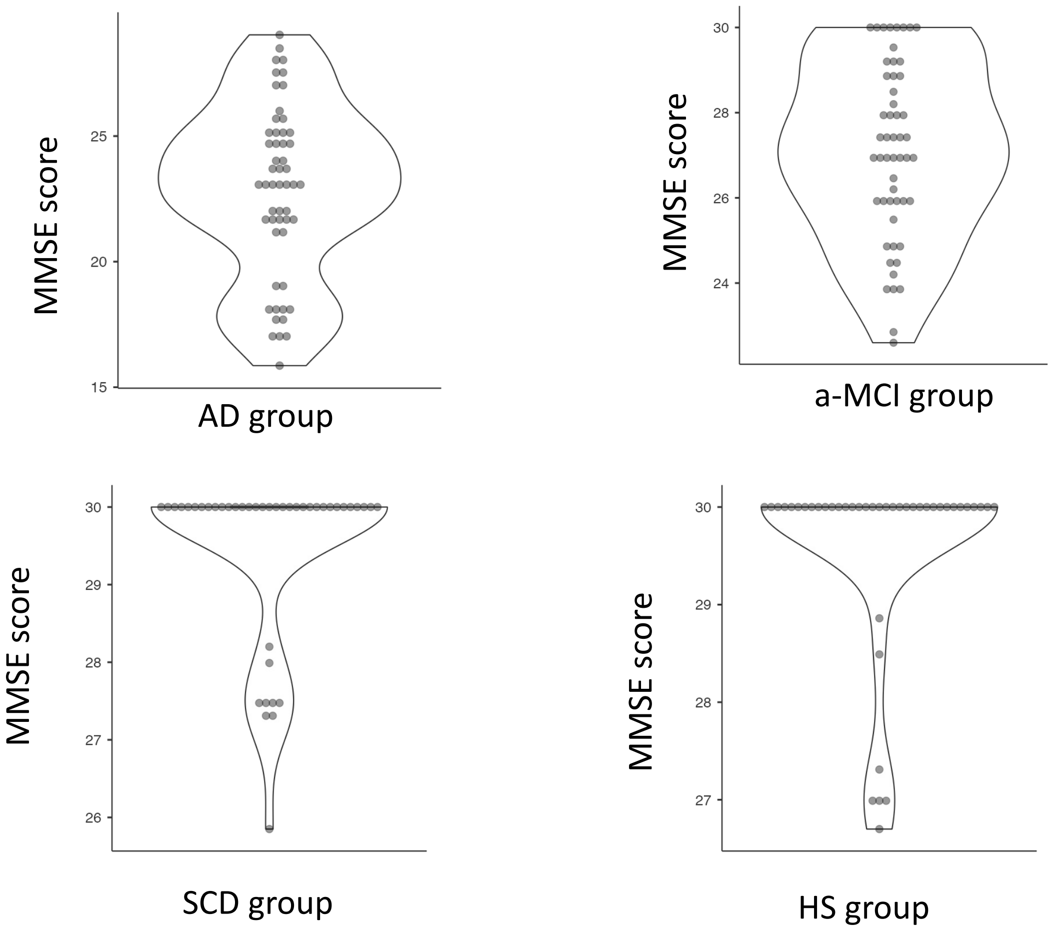

Italian normality cut-off scores > 23.84

Violin plots illustrating the distribution of raw MMSE scores in the Alzheimer's Disease (AD), amnestic Mild Cognitive Impairment (a-MCI), Subjective Cognitive Decline (SCD), and Healthy Subjects (HS) groups. Individual data points are superimposed on each violin. The dashed horizontal line indicates the Italian normative cutoff for cognitive impairment (adjusted score = 23.84; Magni et al., 1996; Measso et al., 1993). Note that the score distributions in the SCD and HS groups are heavily skewed toward the ceiling of the scale (scores of 29-30), reflecting the well-documented ceiling effect of the MMSE in cognitively intact older adults, and that all individuals in these groups scored well above the normative cutoff. Notably, several AD and a-MCI patients, whose diagnosis was neurobiologically confirmed through cerebrospinal fluid biomarkers, obtained MMSE scores above the cutoff threshold, further demonstrating that the MMSE functions as an orientative screening measure rather than a definitive diagnostic criterion, and that group classification in the present study was based on converging clinical, neuropsychological, and biomarker evidence.
